# Synthesis of N-Bridged Pyrido[4,3-*d*]pyrimidines and Self-Assembly into Twin Rosette Cages and Nanotubes in Organic Media

**DOI:** 10.1038/s41598-018-34080-9

**Published:** 2018-10-29

**Authors:** Cansu Igci, Osman Karaman, Yiwen Fan, Arthur A. Gonzales, Hicham Fenniri, Gorkem Gunbas

**Affiliations:** 1 Department of Chemistry, Middle East Technical University, 06800 Ankara, Turkey; 2 Department of Chemical Engineering, Northeastern University, 360 Huntington Avenue, Boston, MA 02115 USA; 3 Department of Bioengineering, Northeastern University, 360 Huntington Avenue, Boston, MA 02115 USA; 4 Department of Chemistry & Chemical Biology, Northeastern University, 360 Huntington Avenue, Boston, MA 02115 USA

## Abstract

Two N-bridged pyrido[4,3-*d*]pyrimidine derivatives were synthesized toward realization of a self-assembled bis-rosette cage, in organic media. Starting from commercially available malononitrile dimer and dimethyl 5-aminoisophthalate, the target molecules were synthesized in 11 steps using a convergent approach. The final bridged compounds were characterized by nuclear magnetic resonance spectroscopy and high-resolution mass spectrometry. The hierarchical self-assembly of the nanocages into rosette nanotubes and nanobundles was established by electron microscopy and molecular modelling studies.

## Introduction

Supramolecular synthesis^1^ became an invaluable tool for the realization of complex molecular architectures that are unimaginable through covalent chemistry. The immense interest around supramolecular chemistry resulted in significant advancements over the years and the prospects for numerous applications are now on the horizon^2–6^. Among the possible non-covalent forces that can be utilized for supramolecular synthesis, H-bonding holds a special place. The strength and directionality of H-bonds have been well-explored in the literature and a variety of structures with different dimensions and shapes have been realized. The early examples from the Whitesides^7–11^, Reinhoudt^12,13^, and Lehn^14^ groups related to cyanuric acid/melamine system demonstrated the immense potential of hydrogen bonding for creating supramolecular assemblies including linear, crinkled, rosette and cage structures. In these assemblies, even though H-bonds were used to encode chemical information, steric and solvophobic effects also played a vital role.

**Figure 1:**
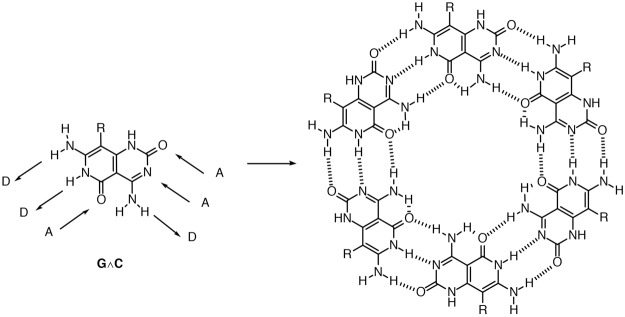
Mascal rosette structure based on the G∧C base motif.

**Figure 2:**
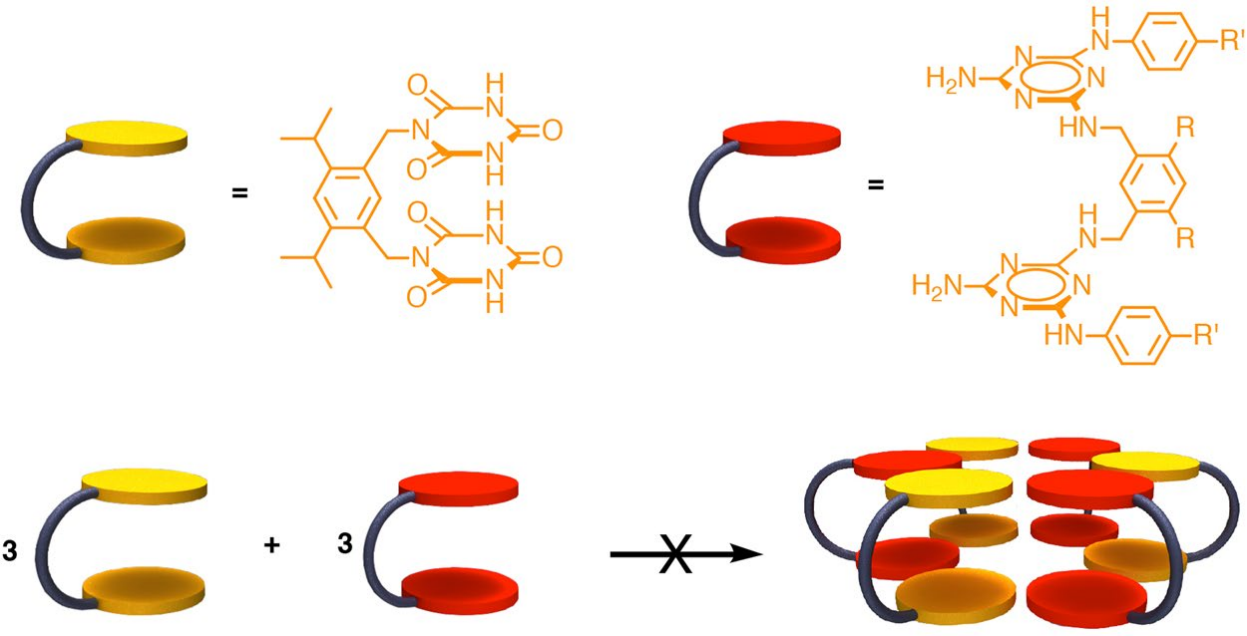
Whiteside approach of bis-rosette cages using cyanuric acid and melamine derivatives^17^.

Mascal^15^ and Lehn^16^ later showed that hexameric rosette structures can be achieved using an DNA-base hydrid (pyrido[4,3-*d*]pyrimidine), which utilize only programmed H-bond information, the G∧C base (Fig. 1). In addition to rosette structure, their cage analogues were also important targets in supramolecular synthesis. A number of examples were realized using cyanuric acid/melamine system. However, Whitesides *et al*. showed that using a bridged cyanuric acid and a bridged melamine derivative did not yield the expected results^17^. It was concluded that due to the electron deficient nature of cyanuric acid, the required eclipsed conformation could not be achieved (Fig. 2).

**Figure 3:**
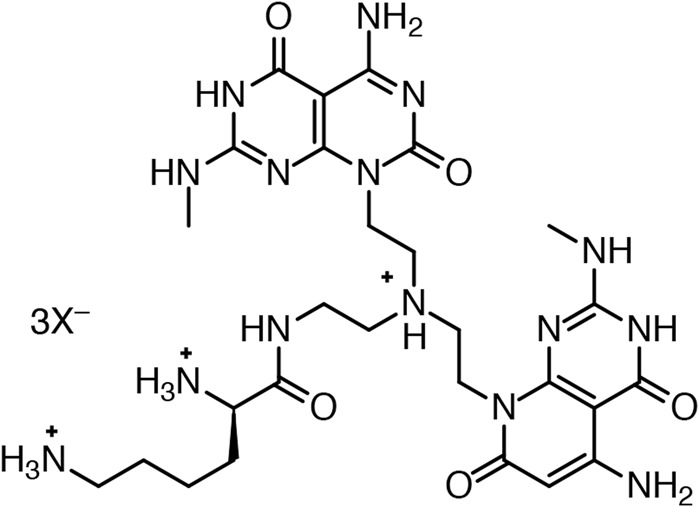
Fenniri’s G∧C motif that self-assembles into a bis-rosette cage^18^.

More recently, Fenniri and co-workers realized a twin-rosette structures utilizing a G∧C-base derivative, which relied on programmed H-bond information to direct the self-assembly while stacking interactions and hydrophobic effect provided the bulk of the energy for this entropically-driven self-assembly process (Fig. 3)^18^.

**Figure 4:**
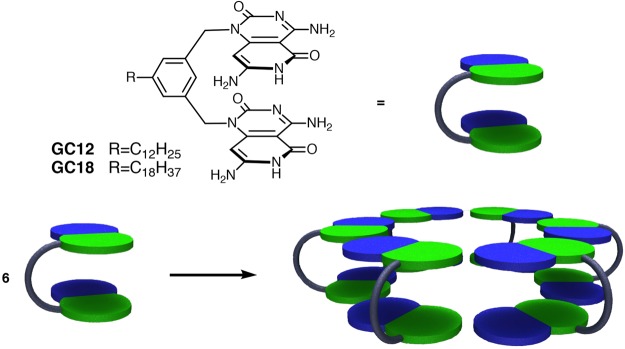
Structures of G∧C bases GC12 and GC18 and schematic representation of the target cage assembly.

In this report, our motivation was to design and synthesize a G∧C-base hybrid which would assemble into a twin-rosette cage structure utilizing only programmed H-bonding information. Additionally, we wanted to establish whether such supramolecular cages could further self-organize into rosette nanotubes (RNTs). To test our hypotheses, we opted to (a) use Mascal’s base due to its ease of synthesis and subsequent synthetic modifications, and (b) use a rigid bridging group to minimize the entropic penalty. With these considerations in mind we designed the bases GC12 and GC18, which were expected to assemble into rosette nano-cages via hydrogen bonding interactions (Fig. 4). Although the distance between the bases within each motif exceeds the van der Waals optimal distance for efficient stacking interactions, we anticipated that a collapse of the benzene ring could bring the bases in a favorable staggered stacking interaction and promote rosette-rosette stacking interactions. The ultimate goal in our research program is to understand the photonic and electronic properties of G∧C-based assemblies in organic media and we believe GC12 and GC18 would be quite important in this endeavor.

## Results and Discussion

**Figure 5:**
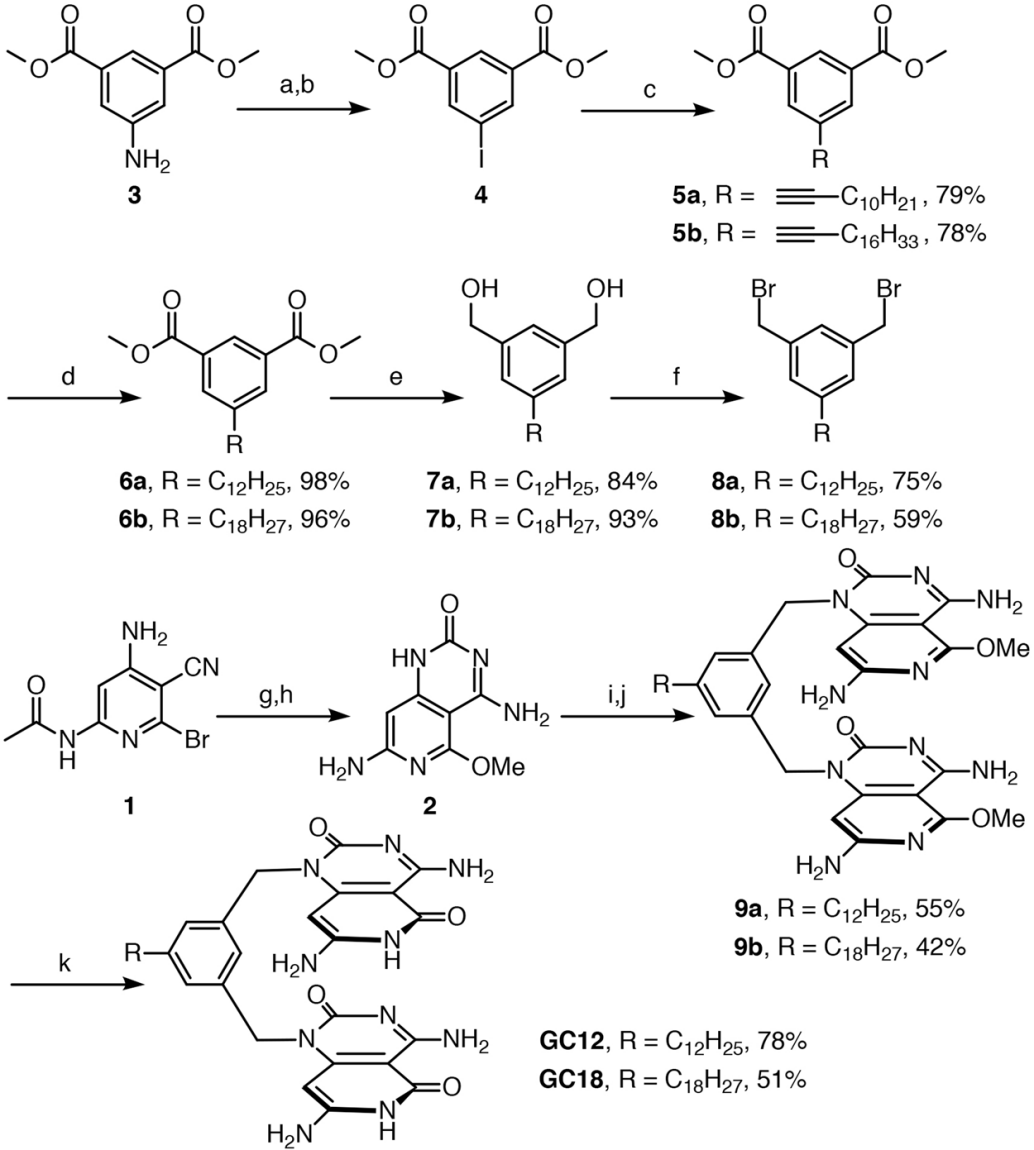
Synthetic pathway for GC12 and GC18. Reagents and conditions: (a) HCl, NaNO_2_, 0 °C, 45 min; (b) KI, H_2_O, CH_2_Cl_2_, rt, 4 h, 58% (2 steps); (c) PdCl_2_(PPh_3_)_2_, CuI, Et_3_N, THF, rt, 1 h; (d) Pd/C, H_2_, CH_3_OH, rt, 18 h; (e) LiAlH_4_, THF, rt, 2 h; (f) 33% HBr/AcOH (w/w), rt, 2 h; (g) Cl_3_CONCO, CH_2_Cl_2_, rt, 48 h; (h) NaOCH_3_, CH_3_OH, reflux, 18 h, 78% (2 steps); (i) *n*-BuLi, THF, −78 °C→−10 °C; (j) 8a or 8b, DMF, rt, 96 h; (k) TMSCl, NaI, CH_3_CN, reflux, 3 h.

Figure 5 shows the synthetic strategy for the target molecules. The synthesis of unreported compounds 8a and 8b featuring C12 and C18 alkyl chains started with commercially available dimethyl 5-aminoisophthalate 3 which was subjected to Sandmeyer reaction conditions to give dimethyl 5-iodoisophthalate 4^19^. Sonagashira coupling^20^ was then performed with two different terminal alkynes to yield compounds 5a and 5b in good yields. Hydrogenation over Pd/C catalyst gave target esters 6a and 6b in excellent yields. Esters 6a and 6b were then reduced to the corresponding alcohol derivatives 7a and 7b with LiAlH_4_. Finally, for the target bridging groups, alcohols 7a and 7b were converted to the dibromo analogues (8a and 8b) with HBr in acetic acid. Compounds 1 and 2 were synthesized according to literature reports with minor modifications to improve product separation^21^. Literature reports^21^ suggest that deprotonation of compound 2 with NaH in DMF, followed by addition of the alkylbromides 8 would give alkylation products 9 in satisfactory yields. However, in our case these conditions gave very low yields. Further review of the literature indicated that NaH reacts with DMF to produce significant amounts of NaNMe_2_, which reacts with alkylbromides 8^22^. In earlier reports^21^, this side reaction went unnoticed since excess alkylbromides were utilized. In our case, however, half an equivalent of the alkylbromide was used for every equivalent of 2. To circumvent this side reaction we first deprotonated compound 2 with *n*-BuLi (1 eq) in THF at −78 °C and the mixture was gradually warmed to −10 °C. Dibromides 8 in DMF was then added and the mixture was stirred at room temperature. Significantly higher yields of 9 were achieved using this approach. Even though there is possibility for both N and O alkylation, we believe N-alkylation is dominant since for similar systems, under similar conditions, x-ray data proved that the alkylation goes on nitrogen^21^. Additionally a number of literature examples have shown that O-alkylation can be achieved if alkylsulfonates are used as electrophile^23^. Alkyl bromides, in most cases, give N-alkylation. The ^1^H NMR shift for N-alkylation versus O-alkylation is also indicative. N-alkylation of cytosine ring with benzyl bromide results in a structure where the CH_2_ resonates at 4.82 ppm^24^. This is close to 5.09 ppm that we observe for our system. Literature reports have also shown that the benzylic CH_2_ of O-alkylated compounds has a chemical shift of 5.76 ppm, which is significantly higher than the 5.09 ppm resonance we observed^25^. After the successful synthesis of 9a and 9b removal of the methyl ether protecting groups was performed using *in situ*-generated TMSI to yield target compounds GC12 and GC18.

**Figure 6:**
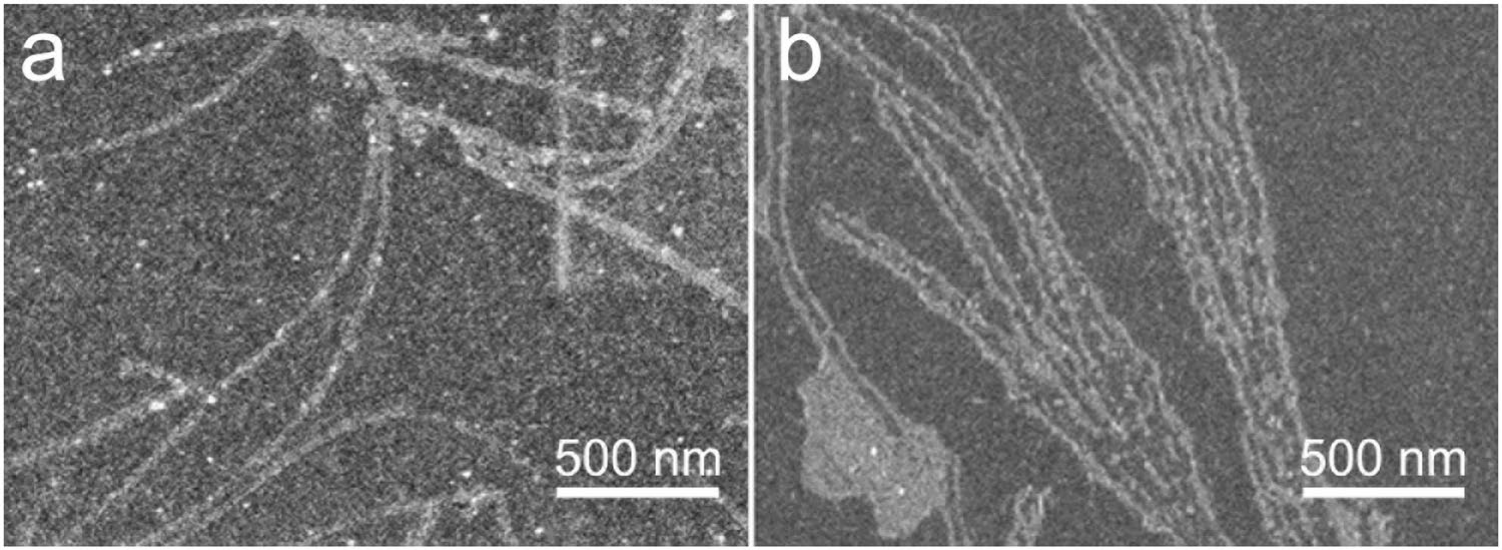
SEM images of GC12 (**a**) and GC18 (**b**) showing the formation of fibrous materials with a diameter of 65.0 ± 2.3 nm and 35.2 ± 5.0 nm from GC12 and GC18, respectively. The samples were prepared as detailed in the supporting information section.

The characterization of the self-assembled structures from GC12 and GC18 using NMR spectroscopy was not successful due to their limited solubility in common organic solvents such as CHCl_3_, DCM MeCN, DMSO. The structural characterization of GC12 and GC18 was achieved by NMR in deuterated trifluoroacetic acid (*d*-TFA) as a solvent, which was expected to prevent hydrogen bonding and subsequent self-assembly. To gain further insight on the self-assembly of these compounds, we turned our attention to electron microscopy and molecular modeling. Scanning electron microscopy (SEM) imaging of both GC12 and GC18 showed the formation of nanobundles with a diameter of 65.0 ± 2.3 nm and 35.2 ± 5.0 nm from GC12 and GC18, respectively (Fig. 6).

**Figure 7:**
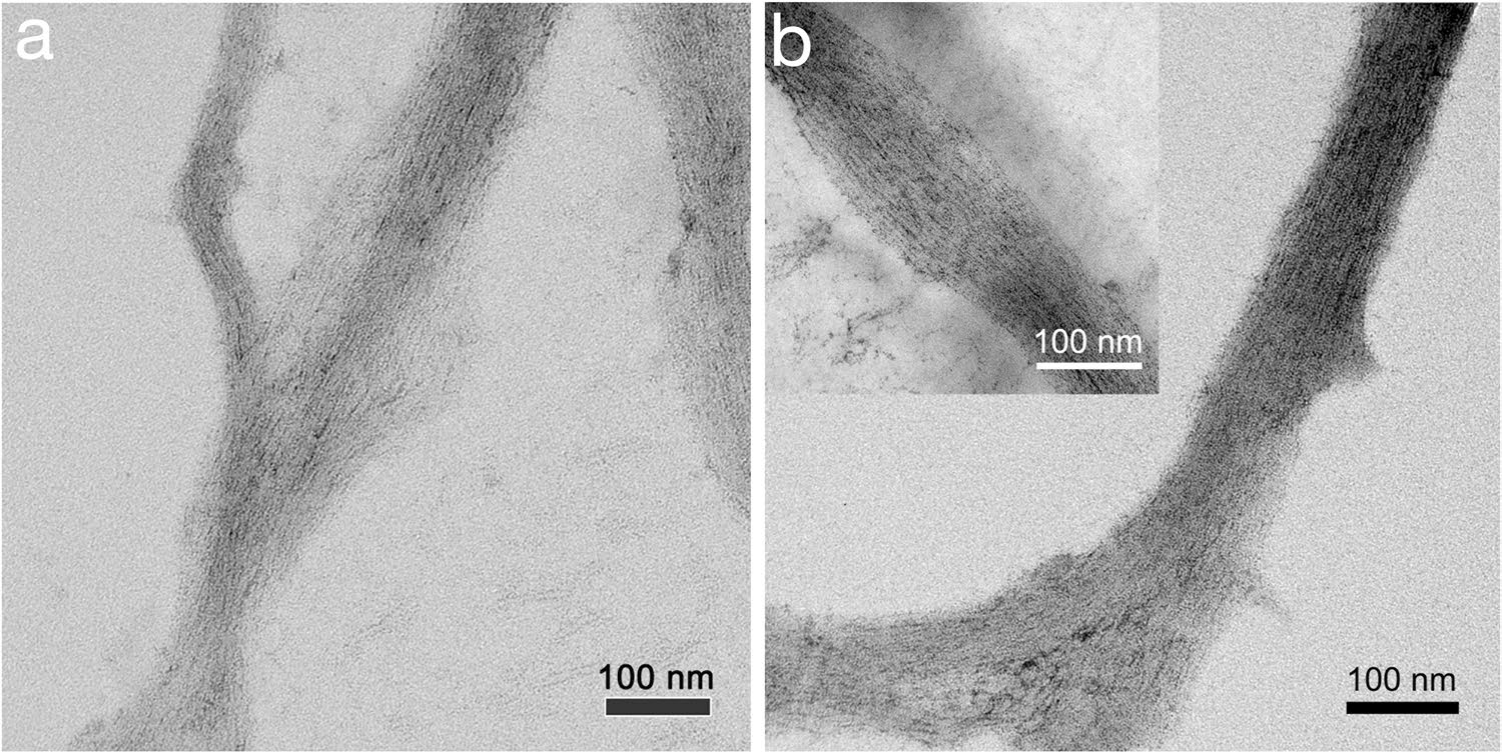
TEM images of nanobundles formed from (**a**) GC12 and (**b**) GC18 showing that they are composed of bundled RNTs.

To gain a better understanding of the nature of the nanobundles observed, transmission electron microscopy (TEM) studies were performed for both GC12 and GC18. From the TEM analyses it became clear that the nanobundles consisted of interwoven RNTs (Fig. 7), which is in agreement with previously reported studies of G∧C base derivatives^21^. In this case in particular, the RNTs form large nanobundles due to strong van der Waals interactions between the long alkyl chains on their surface. From these high resolution TEM images the diameters of individual nanotubes were measured (supporting information). It was found that the diameter of individual nanotubes were 6.0 ± 0.9 nm and 6.9 ± 0.9 nm for GC12 and GC18 respectively. These values are in good agreement with our modeling studies (5.6 nm for GC12 and 6.8 nm for GC18), which are detailed below. GC12 and GC18 were undiscernible by SEM and TEM because their outer surface alkyl chains are equally poor electron scatterers. The difference in dimensions was also undetectable because the effective diameter of both molecules is similar (C12 and C18 have similar effective dimensions).

**Figure 8:**
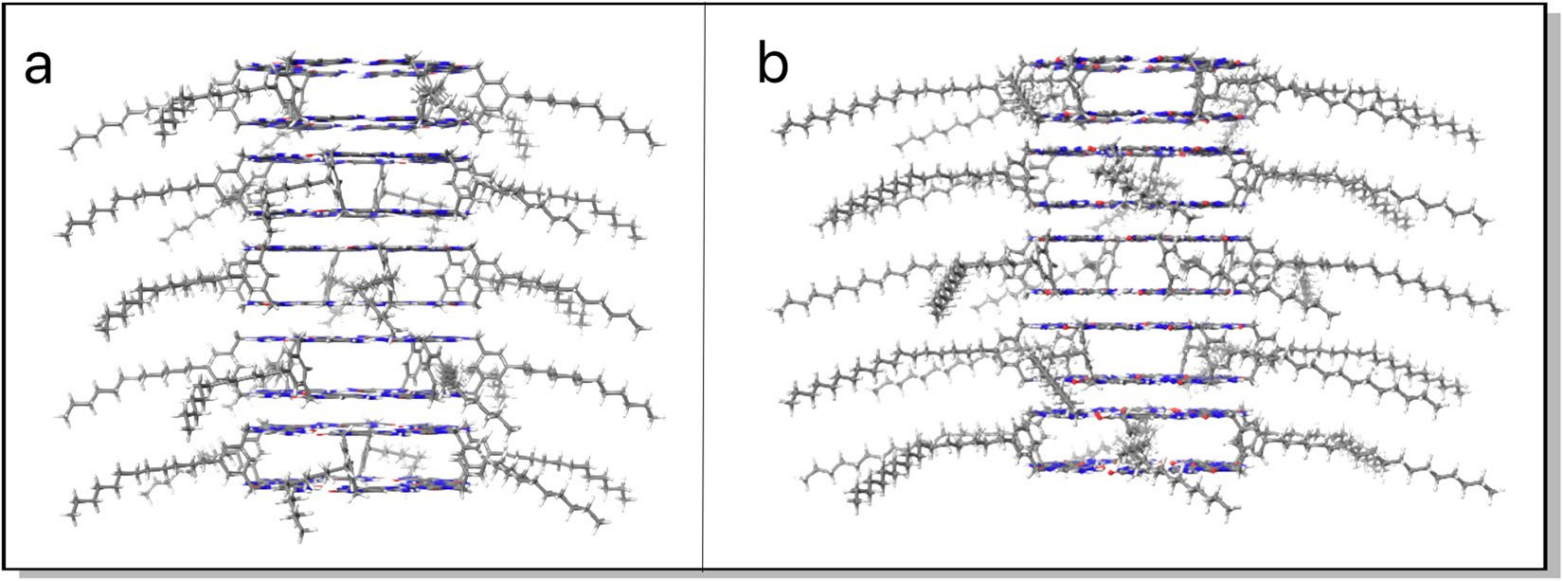
Optimized rosette nanotubes for RNTs derived from GC12 (**a**) and GC18 (**b**).

Computational studies were performed to understand the nature of the assembly and the forces that hold the RNTs. GC12 motif was constructed with an inter-base distance of 4.5 Å and a 6° staggering angle, while the carbon chain was optimized in octanol using Polak-Ribier Conjugate Gradient (PRCG) minimization. The minimized motif was then multiplied and arranged into a hexameric bis-rosette cage stabilized by a network of 36 H-bonds. Five such rosettes were then stacked with an inter-rosette distance of 4.5 Å and a rotation angle of 15° to form a 30-motif rosette nanotube (Figure S1) MD simulations of the RNTs were done in five organic solvents (DMSO, octanol, MeOH, DMF, and cyclohexane) for 50 ns each. The resulting trajectories of the three middle rosettes were then analyzed to obtain nitrogen-nitrogen distance, intra-rosette stacking distance, intra-rosette staggered angle, inter-rosette stacking distance and inter-rosette staggered angle (Figure S2). The definition of these parameters and the obtained values for these parameters from the analyses of the trajectories are given in supporting information (Figure S3). Root mean square deviation calculations of the G∧C bases of the three middle rosettes in all the simulations suggest that the RNTs are stable. The obtained maximum standard deviation of just 0.13 Å of the RNT bases in DMSO indicates this as well (Figure S4). Using the parameter values obtained, the next set of motifs and RNTs were constructed. By again using PRCG minimization, alkyl groups were minimized in octanol to obtain their most probable conformation. During minimization, the top and bottom rosettes as well as all the G∧C bases were fixed to reduce end effects. After minimization, the middle rosette was taken out to construct the final RNT models (Figs 8 and S5).

We then determined the total energy of the RNTs as a function of motif number. RNTs composed of N = 1 to 30 motifs were constructed by symmetric addition of rosettes and stacking to form the nanotube. The association energy, defined as the difference of the total energy of the RNT and the total energy of a single motif multiplied by the number of motifs in the structure, was obtained for each nanotube. Our results suggest that both GC12 and GC18 derived RNTs are stable in solution (Figure S7). The negative trends of both the total energy and association energy further suggest that the formation of nanotubes from free motifs is spontaneous (Figure S6). As can be seen from Fig. 8 the intra-rosette stacking distance is larger compared to inter-rosette stacking distance. However, the stacking interaction throughout the RNT is still strong as suggested by the strong association energy. The ultimate goal in our research program is to understand the photonic and electronic properties of G∧C-based assemblies formed in organic media and we believe GC12 and GC18 with different stacking distances (intra-rosette versus inter-rosette) would provide important insight.

**Figure 9:**
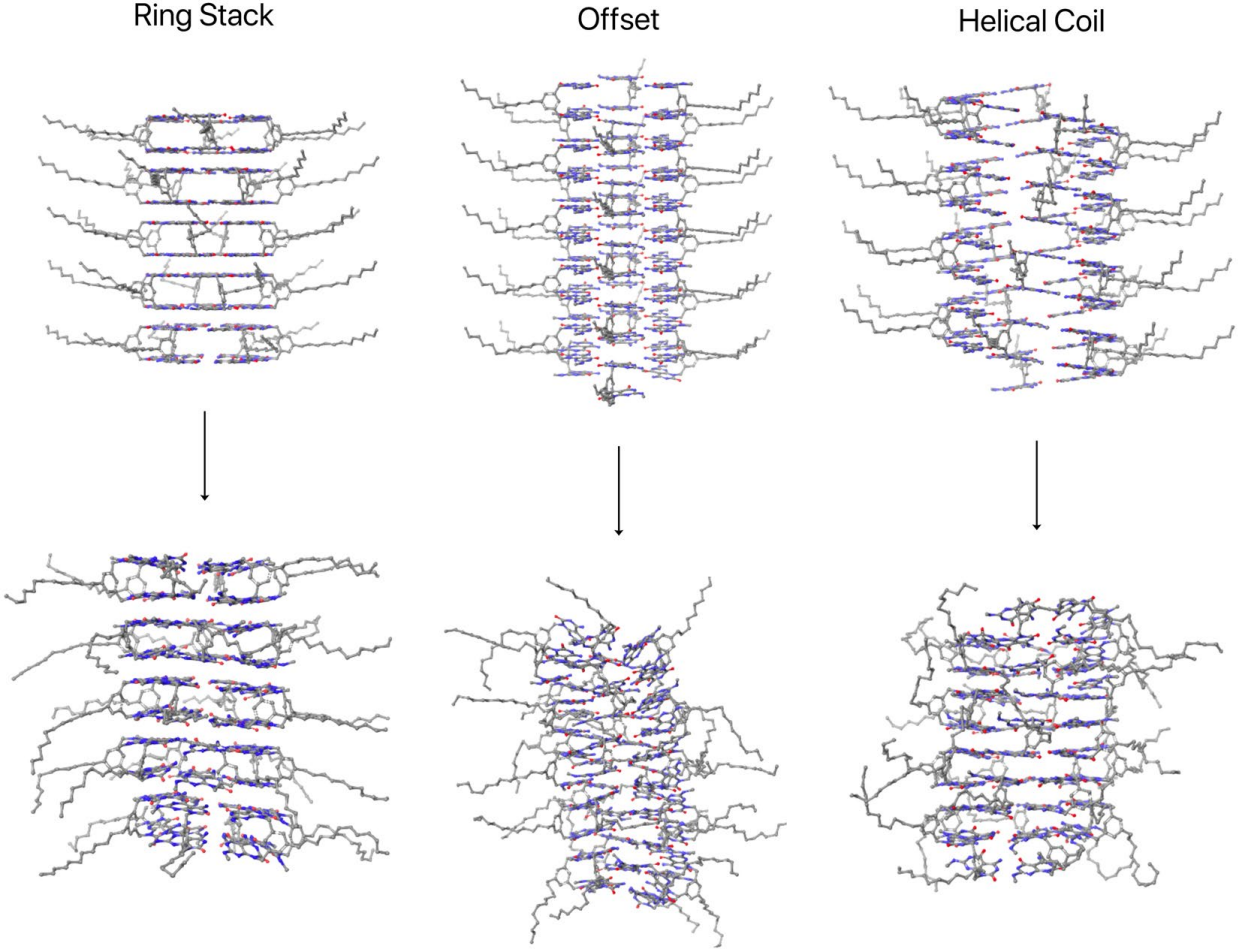
Snapshots from molecular dynamic simulations at the start (0 ns, upper row) and at the end (50 ns, lower row) of the simulation for the stack, offset, helical coil RNT arrangements.

To eliminate other possibilities for the most probable RNT arrangement, MD simulations in DMSO were run using three starting conformations represented by (a) tubular stacks, in which the twin rosettes are stacked on top of each other; (b) offset tubular stacks, in which the twin G∧C bases are shared between two consecutive twin rosettes; and (c) helical coils, in which the twin G∧C bases form a helical arrangement with no separated twin rosettes. Figure 9 shows the before (0 ns) and after (50 ns) snapshots of the three runs. While the ring stack maintained its structure, the offset structure did not hold and has started to transition to ring stacks. The helical coil simply switched to a ring stack conformation after equilibration (around 10 ns).

## Conclusion

In conclusion, a convergent synthetic strategy for the preparation of twin G∧C bases with rigid bridging groups (GC12 and GC18) was successfully devised and the resulting target molecules were carefully characterized. Their subsequent hierarchical self-assembly was further established by electron microscopy and molecular modeling. Our results confirmed our design strategy for a twin rosette cage structure which then form RNTs via π-π stacking interactions and van der Waals interactions. The study highlights the power of programmed H-bonding information in directing the self-assembly of complex architectures in organic media.

## Experimental Section

### Methods

All reagents were commercially available and used without further purification unless otherwise noted. All dry solvents used in reactions were directly used from the Mbraun MBSPS5 solvent drying system ^1^H-NMR and ^13^C-NMR were collected on a Bruker Avance III Ultrashield 400 MHz NMR spectrometer.

### Sample Preparation for SEM and TEM Imaging Studies

GC12 and GC18 were dissolved in dimethylformamide (DMF, 0.4 mg/mL for 1 and 0.2 mg/mL for 2) by sonicating for 30 min at room temperature. The solutions were heated on heating block at 90 °C for 0.5 hour. The result solutions were allowed cool to room temperature followed by 1 day aging. SEM samples were prepared by depositing a droplet of solutions on carbon coated 300 mesh copper grids and blotting after 1 min. All samples were air-dried at least 24 hours prior to imaging. SEM images were obtained without staining at a 5 kV accelerating voltage, 10 μA, and a working distance of 3-4 mm on Hitatch S4800 cold field-emission scanning electron microscope. TEM samples were prepared by depositing the solutions on carbon coated 300 mesh copper grids and blotting after 1 min. The samples were stained by uranyl acetate (2% in water). TEM characterization was performed at 80 kV and 60 μA on JEOL 1010 transmission electron microscope.

### Synthetic Details

Synthetic details of all compounds and their characterization data (including NMR spectra) are given in the Supporting Information. Synthetic details of the final compounds GC12 and GC18 are given below.

### Synthesis of GC12

To a solution of 1,1′-((5-dodecyl-1,3-phenylene)bis(methylene))bis(4,7-diamino-5-methoxy pyrido[4,3-*d*]pyrimidin-2(1 *H*)-one) (60.0 mg, 0.088 mmol) in dry MeCN (14 mL) were added sodium iodide (0.980 g, 0.650 mmol) and chlorotrimethylsilane (0.060 mL, 0.045 g, 0.430 mmol). The reaction flask was protected from light and the mixture was refluxed for 3 h. The mixture was poured into aqueous phosphate buffer (pH 7, 0.5 M, 15 mL). The resulting precipitate was filtered, washed with EtOAc, and then with MeOH to give an off-white solid (45.0 mg, 78%).

### Synthesis of GC18

To a solution of 1,1′-((5-dodecyl-1,3-phenylene)bis(methylene))bis(4,7-diamino-5-methoxy pyrido[4,3-*d*]pyrimidin-2(1 *H*)-one) (80.0 mg, 0.104 mmol) in dry MeCN (16.0 mL), sodium iodide (0.174 g, 1.16 mmol) and chlorotrimethylsilane (0.100 mL, 83.0 mg, 0.766 mmol) were added. The reaction flask was protected from light and the mixture was refluxed for 3 h. The mixture was poured into an aqueous phosphate buffer (pH 7, 0.5 M, 15 mL). The resulting precipitate was filtered, washed with EtOAc, and then with MeOH to give an off-white solid (39.0 mg, 51%).

## Electronic supplementary material

Supporting Information

## Data Availability

The datasets generated during and/or analysed during the current study are available from the corresponding author on reasonable request.
