# Supplementary material for: Synthesis of N-Bridged Pyrido[4,3-d]pyrimidines and Self-Assembly into Twin Rosette Cages and Nanotubes in Organic Media
Source: Sci Rep. 2018 Oct 29;8:15949. doi: 10.1038/s41598-018-34080-9 (PMC6206060; doi:10.1038/s41598-018-34080-9)
Supplement: Supplementary file 1 — Supporting Information [file 41598_2018_34080_MOESM1_ESM.pdf]

SUPPORTING INFORMATION

Synthesis of N-Bridged Pyrido[4,3-*d*]pyrimidines and Self-Assembly into Twin Rosette Cages and Nanotubes in Organic Media

Cansu Igci,[1] Osman Karaman[1], Yiwen Fan,[2] Arthur A. Gonzales, III,[2] Hicham Fenniri,[2,3,4]* Gorkem Gunbas[1,]*

[1]Department of Chemistry, Middle East Technical University, 06800, Ankara, TURKEY. Departments of [2]Chemical Engineering, [3]Bioengineering, [4]Chemistry & Chemical Biology, Northeastern University, 360 Huntington Avenue, Boston, MA 02115, USA.

General

All reagents were commercially available and used without further purification unless otherwise noted. All dry solvents used in reactions were directly used from the Mbraun MBSPS5 solvent drying system ^{1}H-NMR and ^{13}C-NMR were collected on a Bruker Avance III Ultrashield 400 MHz NMR spectrometer.

Sample Preparation for SEM and TEM Imaging Studies

GC12 and **GC18** were dissolved in dimethylformamide (DMF, 0.4 mg/mL for 1 and 0.2 mg/mL for 2) by sonicating for 30 min at room temperature. The solutions were heated on heating block at 90 °C for 0.5 hour. The result solutions were allowed cool to room temperature followed by 1 day aging. SEM samples were prepared by depositing a droplet of solutions on carbon coated 300 mesh copper grids and blotting after 1 min. All samples were air-dried at least 24 hours prior to imaging. SEM images were obtained without staining at a 5 kV accelerating voltage, 10 µA, and a working distance of 3-4 mm on Hitatch S4800 coldfield-emission scanning electron microscope. TEM samples were prepared by depositing the solutions on carbon coated 300 mesh copper grids and blotting after 1 min. The samples were stained by uranyl acetate (2% in water). TEM characterization was performed at 80 kV and 60 µA on JEOL 1010 transmission electron microscope.

Synthetic Details

Synthesis of dimethyl 5-(dodec-1-yn-1-yl)isophthalate (5a). This compound was synthesized according to the literature with small modifications.[1] Dimethyl-5-iodoisophthalate (0.560 g, 1.75 mmol), 1-dodecyne (0.72 g, 0.94 mL, 4.4 mmol), and Et_3N (0.45 g, 0.60 mL, 4.3 mmol) were stirred in dry THF (13 mL) and then, $PdCl_2(PPh_3)_2$ (60 mg, 0.09 mmol) and CuI (30 mg, 0.18 mmol) were added to this solution. The reaction mixture was stirred for 2 h under Argon (Ar) atmosphere at room temperature (rt). The resulting ammonium salt was filtered and the solvent was concentrated under reduced pressure. The residue was purified by silica gel column chromatography (1:6 EtOAc:hexane) to yield target the product as a brown solid (0.495 g, 79%).^{1}H NMR (400 MHz, $CDCl_3$) δ: 8.48 (t, *J* = 1.6 Hz, 1H), 8.14 (d, *J* = 1.6 Hz, 2H), 3.87 (s, 6H), 2.34 (t, *J* = 7.1 Hz, 2H), 1.57–1.50 (m, 2H), 1.42–1.33 (m, 2H), 1.27–1.15 (m, *J* = 11.5 Hz, 12H), 0.80 (t, *J* = 6.8 Hz, 3H). ^{13}C NMR (100 MHz, $CDCl_3$) δ: 164.16, 134.95, 129.13, 127.75, 123.61, 91.23, 77.22, 50.82, 30.30, 27.99, 27.92, 27.72, 27.55, 27.34, 26.96, 21.08, 17.77, 12.49. HRMS calculated for $C_{22}H_{31}O_4$: 359.2222, found: 359.2234.

Synthesis of dimethyl 5-dodecylisophthalate (6a). This compound was synthesized according to the literature with small modifications.[1] To a solution of dimethyl-5-(dodec-1-yn-1-yl)isophthalate (0.440 g, 1.23 mmol) in MeOH (15 mL) Pd/C catalyst (10 wt. %, 0.05 g) was added under Ar atmosphere. Then, the flask was evacuated under vacuum and flushed with H_2 (3x) and the reaction was stirred under a H_2 balloon at rt for 24 h. The mixture was filtered through a bed of Celite, washed with MeOH, and concentrated to yield the target product as a white solid (0.440 g, 98%).

^{1}H NMR (400 MHz, $CDCl_3$) δ: 8.43 (t, *J* = 1.6 Hz, 1H), 7.97 (d, *J* = 1.6 Hz, 2H), 3.87 (s, 6H), 2.63 (t, *J* = 7.7 Hz, 2H), 1.62–1.50 (m, 2H), 1.29–1.15 (m, 18H), 0.80 (t, *J* = 6.8 Hz, 3H). ^{13}C NMR (100 MHz, $CDCl_3$) δ: 166.50, 143.82, 133.83, 130.52, 128.18, 52.27, 35.58, 31.91,

31.24, 29.62, 29.53, 29.42, 29.34, 29.18, 22.68, 14.09. HRMS calculated for $C_{22}H_{35}O_4$: 363.2535, found: 363.2538.

Synthesis of (5-dodecyl-1,3-phenylene)dimethanol (7a). This compound was synthesized according to the literature with small modifications.[2] Dimethyl 5-dodecylisophthalate (0.217 g, 0.599 mmol) dissolved in anhydrous THF (50 mL) and the solution was added dropwise to the a cooled (0 °C) solution of lithium aluminum hydride (76.0 mg, 2.00 mmol) in anhydrous THF (3 mL). The mixture was stirred at rt for 2 h. Deionized water (dH_2O, 0.1 mL) and 15% (w/w) NaOH solution (0.1 mL) were sequentially added to the resulting mixture to quench reaction. The residue was filtered and washed with THF (20 mL), then concentrated to obtain a white solid (0.154 g, 84%). ^{1}H NMR (400 MHz, $CDCl_3$) δ: 7.09 (app. s, 1H), 7.02 (app. s, 2H), 4.57 (s, 4H), 2.52 (t, *J* = 7.7 Hz, 2H), 1.96 (app. br. s, 2H), 1.58–1.48 (m, 2H), 1.24–1.17 (m, 18H), 0.81 (t, *J* = 6.8 Hz, 3H). ^{13}C NMR (100 MHz, $CDCl_3$) δ: 141.75, 139.10, 124.33, 120.86, 63.26, 33.84, 29.86, 29.46, 27.62, 27.59, 27.53, 27.46, 27.35, 27.30, 20.63, 12.07. HRMS calculated for $C_{20}H_{34}O_2Na$: 329.2457, found: 329.2466.

Synthesis of 1,3-bis(bromomethyl)-5-dodecylbenzene (8a). This compound was synthesized according to the literature with small modifications.[3] A mixture of (5-dodecyl-1,3-phenylene)dimethanol (0.160 g, 0.520 mmol) and 33% HBr in acetic acid (2 mL) was stirred at rt for 2 h. After addition of DCM (10 mL), the organic solvent was washed with dH_2O and saturated bicarbonate solution and dried over magnesium sulfate. The solvent was evaporated under reduced pressure, and the crude product was purified by silica gel column chromatography (1:4 EtOAc/hexane) to yield white solid (0.168 g, 75%). ^{1}H NMR (400 MHz, $CDCl_3$) δ: 7.16 (t, *J* = 1.5 Hz, 1H), 7.06 (d, *J* = 1.4 Hz, 2H), 4.38 (s, 4H), 2.51 (t, *J* = 7.6 Hz, 2H), 1.59 – 1.48 (m, 2H), 1.30 – 1.13 (m, 18H), 0.81 (t, *J* = 6.8 Hz, 3H).^{13}C NMR (100 MHz, $CDCl_3$) δ: 144.35, 138.23, 129.22, 126.93, 35.64, 33.11, 31.93, 31.20, 29.67, 29.65, 29.57, 29.45, 29.36, 29.31, 22.70, 14.12. HRMS calculated for $C_{20}H_{32}Br_2$: 432.0850, found: 432.0821.

Synthesis of 1,1'-((5-dodecyl-1,3-phenylene) bis(methylene))bis(4,7-diamino-5-methoxypyrido [4,3-*d*]pyrimidin-2(1*H*)-one) (9a). To a solution of 4,7-Diamino-5-methoxypyrido[4,3-*d*]pyrimidin-2(1*H*)-one (**2**, 0.213 g, 1.03 mmol) in dry THF (3 mL), 2.5 M *n*-butyllithium (0.450 mL, 1.13 mmol, in hexanes) was added dropwise at -78 °C, and the mixture was stirred until -10 °C was reached. Then, 1,3-bis(bromomethyl)-5-dodecylbenzene (0.216 g, 0.500 mmol) in dry DMF (25.0 mL) were added, and the mixture was stirred for 4 days. TLC showed ~80% completion according spot size and intensity under UV light. The reaction was quenched with MeOH (20 mL), and concentrated under reduced pressure. The resulting residue was purified by silica gel column chromatography (1:9 MeOH:DCM, two times due to coelution with **2**) to yield an off-white solid (total, 0.190 g, 55%). Rf(**9a**): 0.4, Rf(**2**): 0.1 ^{1}H NMR (400 MHz, DMSO-*d₆*) δ: 7.62 (s, 2H), 7.37 (s, 2H), 6.97 (app. s, 1H), 6.72 (app. s, 2H), 6.66 (s, 4H), 5.61 (s, 2H), 5.02 (br s, 4H), 3.93 (s, 6H), 2.40 (t, *J* = 7.7 Hz, 2H), 1.44–1.32 (m, 2H), 1.25–1.11 (m, 18H), 0.84 (t, *J* = 6.8 Hz, 3H). ^{13}C NMR (100 MHz, DMSO-d_6) δ: 161.61, 161.33, 160.27, 155.34, 151.99, 142.74, 137.27, 124.22, 122.36, 84.77, 82.44, 53.71, 45.98, 34.95, 31.25, 30.81, 28.98, 28.93, 28.88, 28.74, 28.65, 28.48, 22.04, 13.90. HRMS calculated for $C_{36}H_{49}N_{10}O_4$: 685.3938, found: 685.3939.

Synthesis of 1,1'-((5-dodecyl-1,3-phenylene)bis(methylene)) bis(4,7-diaminopyrido [4,3-*d*]pyrimidine-2,5(1H,6H)-dione) (GC12). To a solution of 1,1'-((5-dodecyl-1,3-phenylene)bis(methylene))bis(4,7-diamino-5-methoxy pyrido[4,3-*d*]pyrimidin-2(1*H*)-one) (60.0 mg, 0.088 mmol) in dry MeCN (14 mL) were added sodium iodide (0.974 g, 0.650 mmol) and chlorotrimethylsilane (0.060 mL, 0.045 g, 0.430 mmol). The reaction flask was protected from light and the mixture was refluxed for 3 h. The mixture was poured into aqueous phosphate buffer (pH 7, 0.5 M, 15 mL). The resulting precipitate was filtered, washed with EtOAc, and then with MeOH to give an off-white solid (45.0 mg, 78%). ^{1}H NMR (400 MHz, DMSO-d_6,1 drop of *d*-TFA) δ: 11.87 (s, 2H), 9.69 (s, 2H), 8.62 (s, 2H), 7.08 (app. s, 1H), 7.05 (app. s., 2H), 5.44 (s, 2H), 5.09 (s, 4H), 1.58–1.51 (m, 2H), 1.28 (app. br s, 18H), 0.91 (t, *J* = 6.0 Hz, 3H), (two amine protons overlap with TFA peak).^{13}C NMR (100 MHz, DMSO-d_6) δ: 161.31, 156.28, 155.82, 152.29, 147.96, 143.55, 135.34, 125.13, 82.72, 77.90, 46.30, 34.84, 31.19, 30.82, 28.92, 28.75, 28.59, 21.97, 13.70. HRMS calculated for $C_{34}H_{45}N_{10}O_4$: 657.3625, found: 657.3600.

Synthesis of Dimethyl 5-(octadec-1-yn-1-yl)isophthalate (5b). This compound was synthesized according to a literature procedure.[1] Dimethyl 5-iodoisophthalate (1.03 g, 3.21 mmol), 1-octadecyne (2.01 g, 2.50 mL, 8.03 mmol), and Et_3N (0.81 g, 1.1 mL, 8.0 mmol) was stirred in dry THF (25 mL) and then, $PdCl_2(PPh_3)_2$ (0.11 g, 0.16 mmol) and CuI (60 mg, 0.32 mmol) were added. The reaction mixture was stirred for 2 h under Ar atmosphere at rt. The resulting ammonium salt was filtered and the solvent was concentrated under reduced pressure. The residue was purified by silica gel column chromatography (1:6 EtOAc:hexane) to yield a brown solid (1.11 g, 78%). ^{1}H NMR (400 MHz, $CDCl_3$) δ: 8.56 (t, *J* = 1.6 Hz, 1H), 8.22 (d, *J* = 1.6 Hz, 2H), 3.94 (s, 6H), 2.42 (t, *J* = 7.1 Hz, 2H), 1.68 – 1.55 (m, 2H), 1.51–1.38 (m, 2H), 1.34–1.22 (m, 24H), 0.88 (t, *J* = 6.8 Hz, 3H). ^{13}C NMR (100 MHz, $CDCl_3$) δ: 165.76, 136.56, 130.72, 129.36, 125.20, 92.83, 78.81, 52.44, 31.93, 29.70, 29.67, 29.53, 29.37, 29.17, 28.95, 28.56, 22.70, 19.37, 14.12. HRMS calculated for $C_{28}H_{43}O_4$: 443.3161, found: 443.3156.

Synthesis of Dimethyl 5-octadecylisophthalate (6b). This compound was synthesized according to a literature procedure.[1] To a solution of dimethyl-5-(octadec-1-yn-1-yl)isophthalate (1.12 g, 2.53 mmol) in MeOH (25 mL), Pd/C catalyst (10 wt%, 0.110 g) was added under Ar atmosphere. The flask was then evacuated under vacuum and flushed with H_2 (3x) and the reaction was stirred under H_2 atmosphere (balloon) at rt for 24 h. The mixture was filtered through a bed of celite, washed with MeOH, and concentrated to yield the target compound as an off-white solid (1.08 g, 96%). ^{1}H NMR (400 MHz, $CDCl_3$) δ: 8.43 (app. s, 1H), 7.97 (app. s, 2H), 3.87 (s, 6H), 2.63 (t, *J* = 7.7 Hz, 2H), 1.62–1.50 (m, 2H), 1.28–1.11 (m, 30H), 0.80 (t, *J* = 6.8 Hz, 3H). ^{13}C NMR (100 MHz, $CDCl_3$) δ: 166.50, 143.82, 133.83, 130.51, 128.18, 52.27, 35.58, 31.93, 31.25, 29.70, 29.66, 29.54, 29.43, 29.36, 29.19, 22.68, 14.10. HRMS calculated for $C_{28}H_{47}O_4$: 447.3474, found: 447.3479.

Synthesis of (5-octadecyl-1,3-phenylene)dimethanol (7b). This compound was synthesized according to a literature procedure.[2] Dimethyl 5-octadecylisophthalate (1.09 g, 2.44 mmol) dissolved in anhydrous THF (60 mL) and the solution was added dropwise to a solution of lithium aluminum hydride (0.310 g, 8.17 mmol) in anhydrous THF (12.0 mL) cooled

to 0°C. The mixture was stirred at rt for 2 h. Deionized water (dH_2O, 0.3 mL) and 15% (w/w) NaOH solution (0.3 mL) were sequentially added to the resulting mixture to quench reaction. The residue was filtered and washed with THF (80 mL), then concentrated to obtain a white solid (0.887 g, 93%).^{1}H NMR (400 MHz, $CDCl_3$) δ: 7.12 (app. s, 1H), 7.05 (app. s, 2H), 4.61 (s, 4H), 2.54 (t, *J* = 7.6 Hz, 2H), 1.63 (br. s, 2H), 1.57–1.49 (m, 2H), 1.27–1.15 (m, 30H), 0.81 (t, *J* = 6.8 Hz, 3H). ^{13}C NMR (100 MHz, $CDCl_3$) δ: 143.85, 141.19, 126.40, 122.91, 65.37, 35.91, 31.92, 31.49, 30.32, 29.69, 29.68, 29.66, 29.59, 29.51, 29.41, 29.35, 22.68, 14.10. HRMS calculated for $C_{26}H_{46}O_2Na$: 413.3396, found: 413.3416.

Synthesis of 1,3-bis(bromomethyl)-5-octadecylbenzene (8b). This compound was synthesized according to a literature procedure.[3] A mixture of (5-octadecyl-1,3-phenylene)dimethanol (0.887 g, 2.27 mmol) and 33% HBr in acetic acid (6 mL) was stirred at rt for 2 h. After addition of DCM (10 mL), the organic solvent was washed with dH_2O and saturated sodium bicarbonate solution, and dried over magnesium sulfate. The solvent was evaporated under reduced pressure, and the crude product was purified by silica gel column chromatography (1:4 EtOAc:hexane) to yield a white solid (0.689 g, 59%).^{1}H NMR (400 MHz, $CDCl_3$) δ: 7.16 (t, *J* = 1.7 Hz, 1H), 7.07 (d, *J* = 1.7 Hz, 2H), 4.38 (s, 4H), 2.51 (t, *J* = 7.8 Hz, 2H), 1.57–1.49 (m, 2H), 1.27–1.15 (m, 30H), 0.81 (t, *J* = 6.8 Hz, 3H). ^{13}C NMR (100 MHz, $CDCl_3$) δ: 144.34, 138.24, 129.21, 126.92, 35.64, 33.08, 31.93, 31.18, 29.70, 29.67, 29.57, 29.45, 29.36, 29.31, 22.69, 14.11. HRMS calculated for $C_{26}H_{44}Br_2$: 539.1687, found: 539.1716.

Synthesis of 1,1'-((5-octadecyl-1,3-phenylene)bis(methylene))bis(4,7-diamino-5-methoxypyrido[4,3-*d*]pyrimidin-2(1*H*)-one) (9b). To a solution of 4,7-Diamino-5-methoxypyrido[4,3-*d*]pyrimidin-2(1*H*)-one (0.200 g, 0.970 mmol) in dry THF (3 mL), 2.5 M *n*-Butyllithium (0.400 mL, 1.06 mmol, in hexanes) was added dropwise at -78 °C, and the mixture was stirred until -10 °C was reached. Then, 1,3-bis(bromomethyl)-5-octadecylbenzene (0.240 g, 0.460 mmol) in dry DMF (30 mL) was added, and the mixture was stirred for 4 days. TLC showed ~80% completion according spot size and intensity under UV light. The reaction was quenched with MeOH (20 mL), and concentrated under reduced pressure. The resulting residue was purified by silica gel column chromatography (1:9 MeOH:DCM, 2 times due to coelution with **2**) to yield an off-white solid (total 0.150 g, 42%). Rf(**9b**): 0.3, Rf(**2**): 0.1 ^{1}H NMR (400 MHz, DMSO-d_6) δ: 7.65 (s, 2H), 7.41 (s, 2H), 6.97 (app. s, 1H), 6.72 (app. s, 2H), 6.66 (s, 4H), 5.61 (s, 2H), 5.03 (br. s, 4H), 3.93 (s, 6H), 2.41 (t, *J* = 7.4 Hz, 2H), 1.43–1.34 (m, 2H), 1.29–1.12 (m, 30 H), 0.85 (t, *J* = 6.3 Hz, 3H). ^{13}C NMR (100 MHz, DMSO-d_6) δ: 161.60, 161.31, 160.27, 155.40, 151.93, 142.71, 137.25, 124.22, 122.42, 84.74, 82.45, 53.71, 45.96, 34.95, 31.24, 30.82, 28.97, 28.94, 28.89, 28.75, 28.64, 28.49, 22.04, 13 .90. HRMS calculated for $C_{42}H_{61}N_{10}O_4$: 769.4877, found: 769.4841.

Synthesis of 1,1'-((5-octadecyl-1,3-phenylene)bis(methylene))bis(4,7-diaminopyrido [4,3-*d*]pyrimidine-2,5(1*H*,6*H*)-dione) (GC18). To a solution of 1,1'-((5-dodecyl-1,3-phenylene)bis(methylene))bis(4,7-diamino-5-methoxy pyrido[4,3-*d*]pyrimidin-2(1*H*)-one) (80.0 mg, 0.104 mmol) in dry MeCN (16.0 mL), sodium iodide (0.174 g, 1.16 mmol) and chlorotrimethylsilane (0.100 mL, 83.0 mg, 0.766 mmol) were added. The reaction flask was protected from light and the mixture was refluxed for 3 h. The mixture was poured into an aqueous phosphate buffer (pH 7, 0.5 M, 15 mL). The resulting precipitate was filtered, washed

with EtOAc, and then with MeOH to give an off-white solid (39.0 mg, 51%). ^{1}H NMR (400 MHz, DMSO-d_6, 3 drop of *d*-TFA) δ: 11.88 (s, 2H), 9.65 (s, 2H), 8.81 (s, 2H), 7.03 (app. s, 1H), 6.98 (app. s, 2H), 5.39 (s, 2H), 5.02 (s, 2H), 1.48 (br s, 2H), 1.22 (br s, 30H), 0.84 (br s, 3H), (two amine protons overlap with TFA peak). ^{13}C NMR (100 MHz, DMSO-d_6) δ: 161.30, 156.25, 155.79, 152.28, 147.96, 143.49, 135.34, 125.09, 121.55, 82.76, 77.91, 46.34, 34.86, 31.24, 30.86, 28.97, 28.86, 28.80, 28.65, 28.60, 23.04, 13.88. HRMS calculated for $C_{40}H_{57}N_{10}O_4$: 741.4564, found: 741.4566.

Supporting Figures.

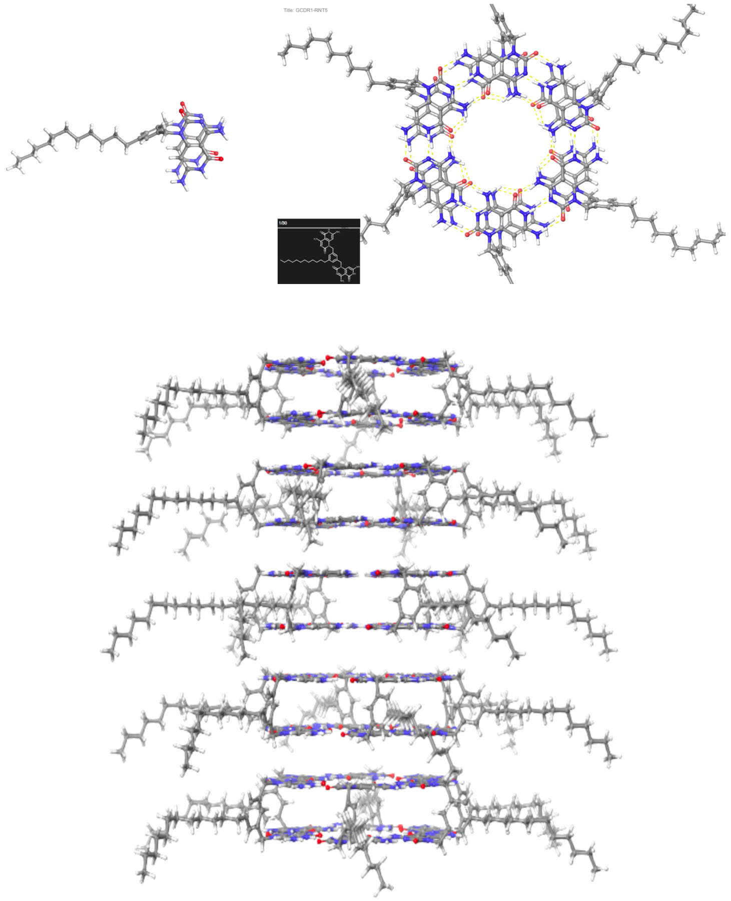

Figure S1. Construction of initial **GC12** motif was achieved using twin G∧C motif with an inter-rosette stacking distance of 4.5 Å and a staggering angle of 6°. For each species, the carbon chain was minimized in octanol in MacroModel software package using Polak-Ribier Conjugate Gradient (PRCG) minimization. The minimized motif was then multiplied and arranged into a hexameric twin rosette cage maintained by a network of 36 hydrogen bonds. Five such rosettes were then stacked with an inter-rosette distance of 4.5 Å and a rotation angle of 15° to form a 60-motif RNT.

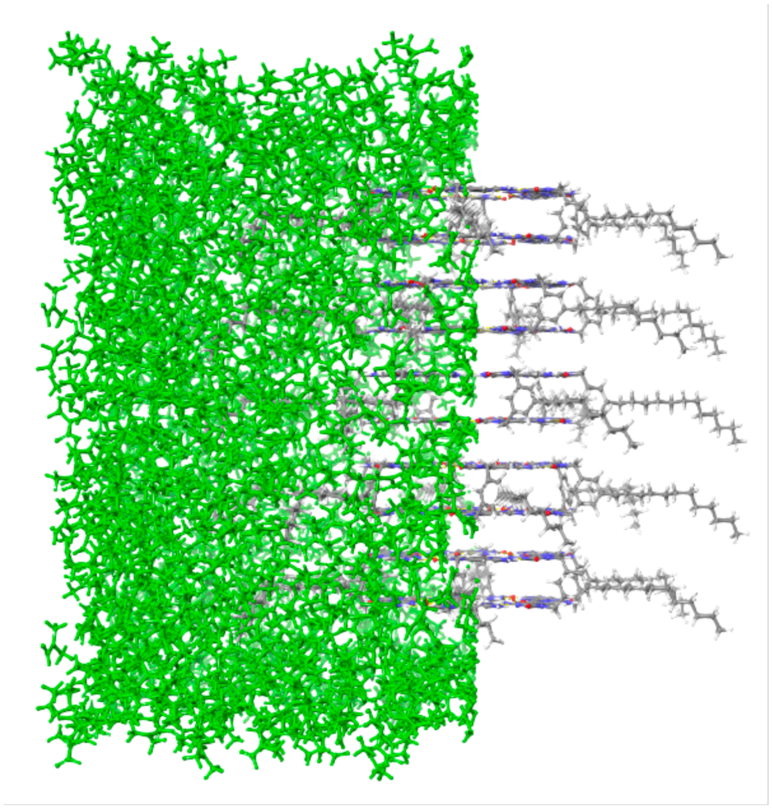

Figure S2. Energy minimization of the RNT using MD simulations. Using Desmond Molecular Dynamics software in Schrödinger Materials Science Suite, MD simulations of the RNTs were done in five organic solvents (DMSO, octanol, methanol, DMF, and cyclohexane). The RNTs were placed in a simulation cell with a 10 Å buffer of solvent. The simulations were run at 300 K and 1 atm for 50 ns each.

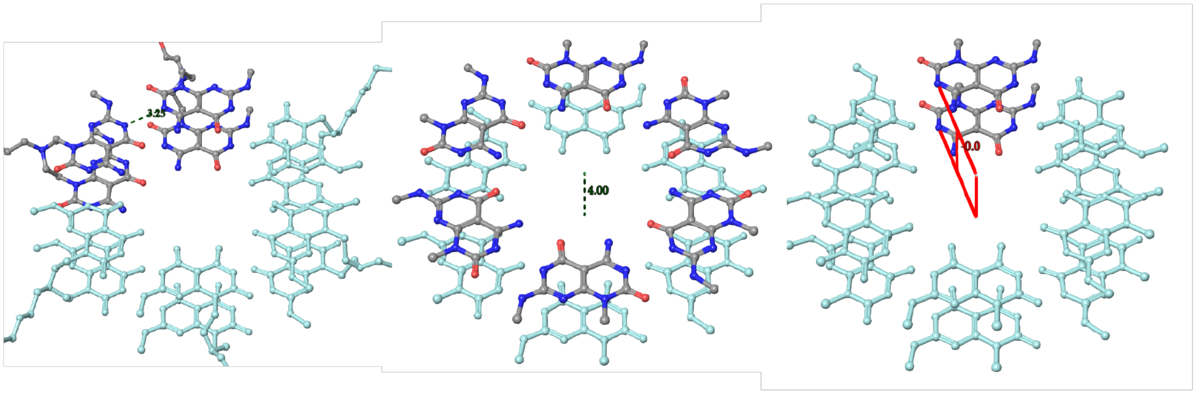

Nitrogen-Nitrogen distance   Intra-rosette stacking distance   Intra-rosette staggered angle

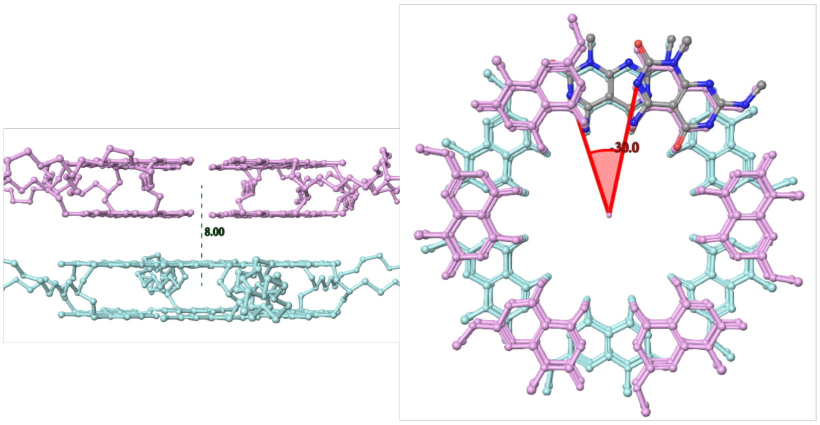

Intra-rosette stacking distance   Intra-rosette staggered angle

Figure S3. Definition of parameters used to construct the RNT and the corresponding values obtained from MD simulations trajectories: Nitrogen-to-nitrogen distance = 2.98 Å; intra-rosette stacking distance = 4.95 Å, intra-rosette staggered angle = 6.12°; inter-rosette stacking distance = 8.15 Å, inter-rosette staggered angle = -38.08°.

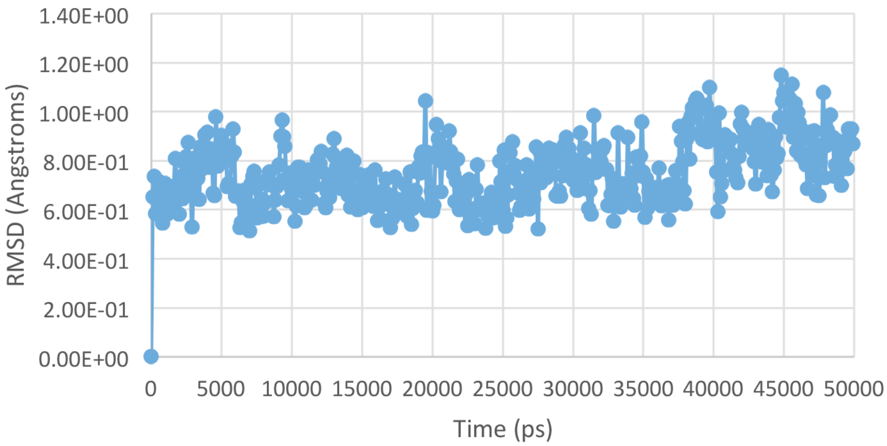

Figure S4. RMSD of the heavy atoms of the G∧C bases in the middle three rosettes of RNTs assembled from GC12.

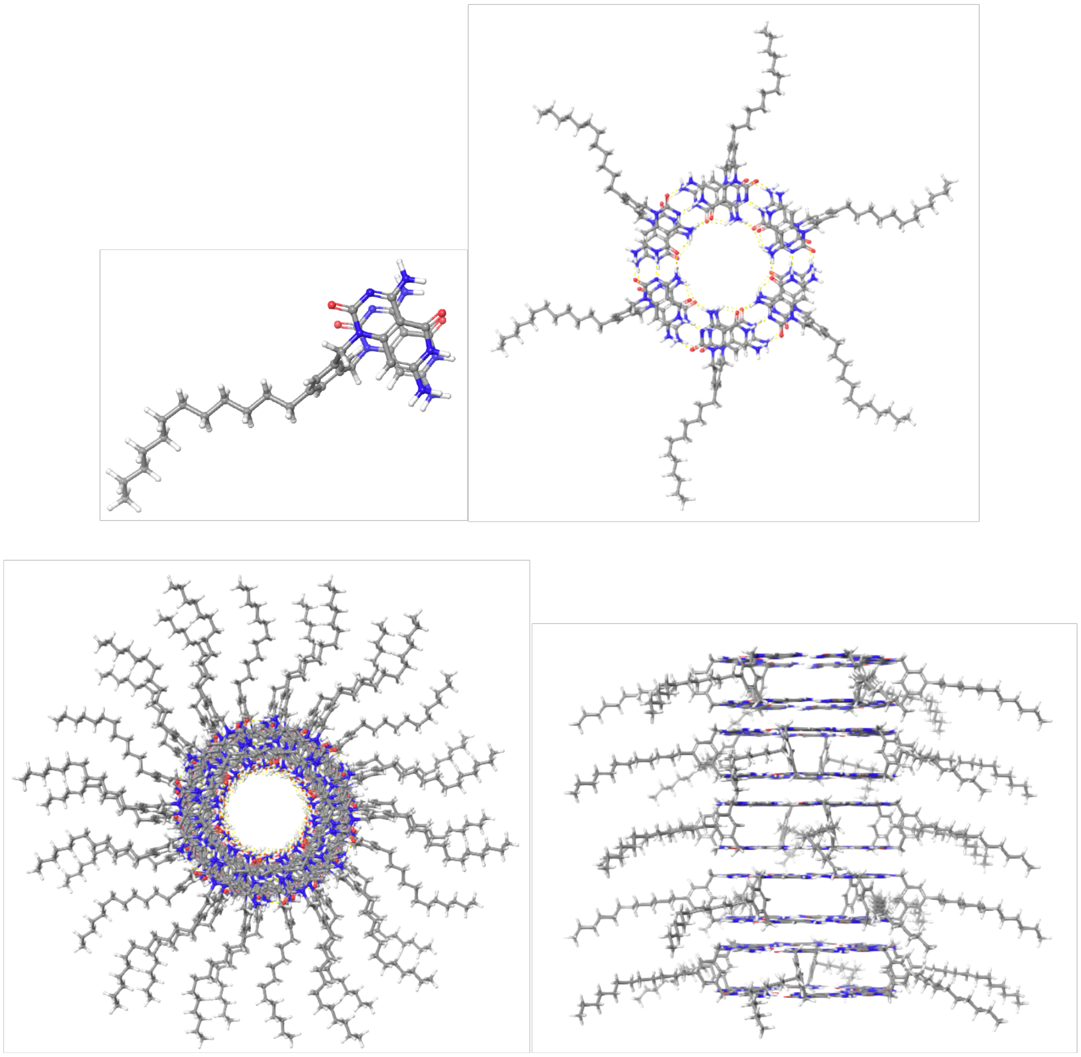

Figure S5. Final GC12 motif and RNTs obtained from GC12.

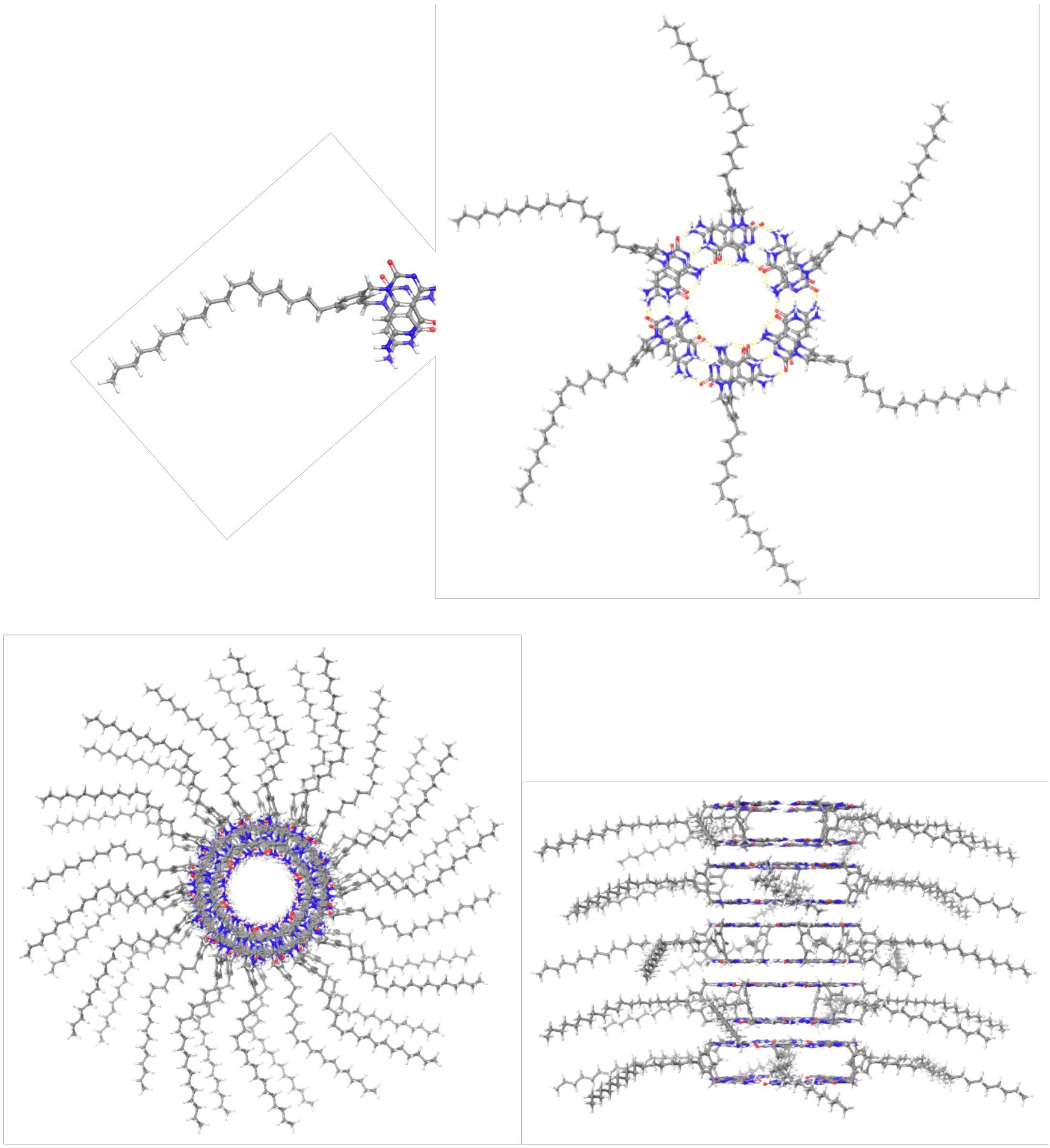

Figure S6. Final GC18 motif and RNTs obtained from GC18.

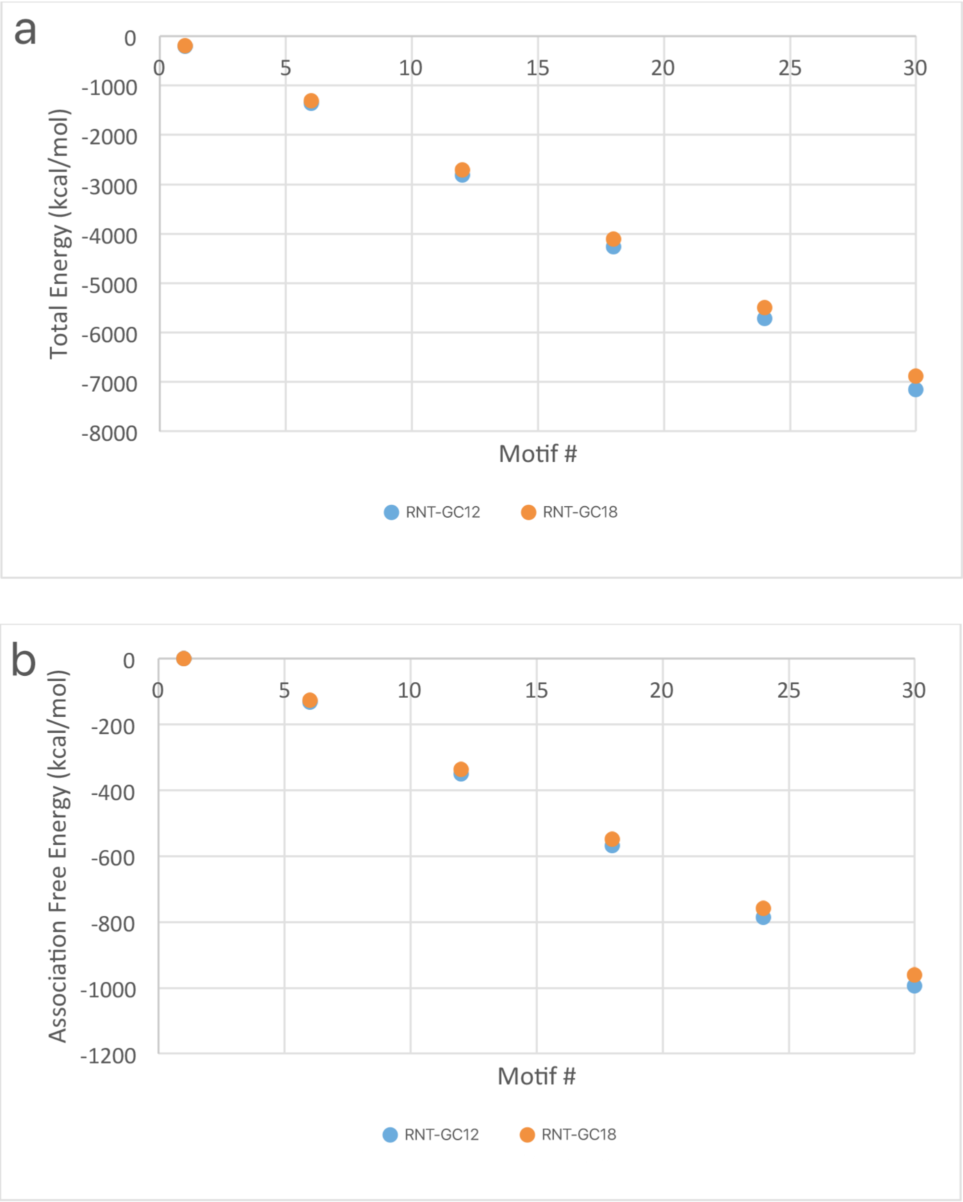

Figure S7. a) Total energy as a function of motif number **b)** and association free energy as a function of motif number.

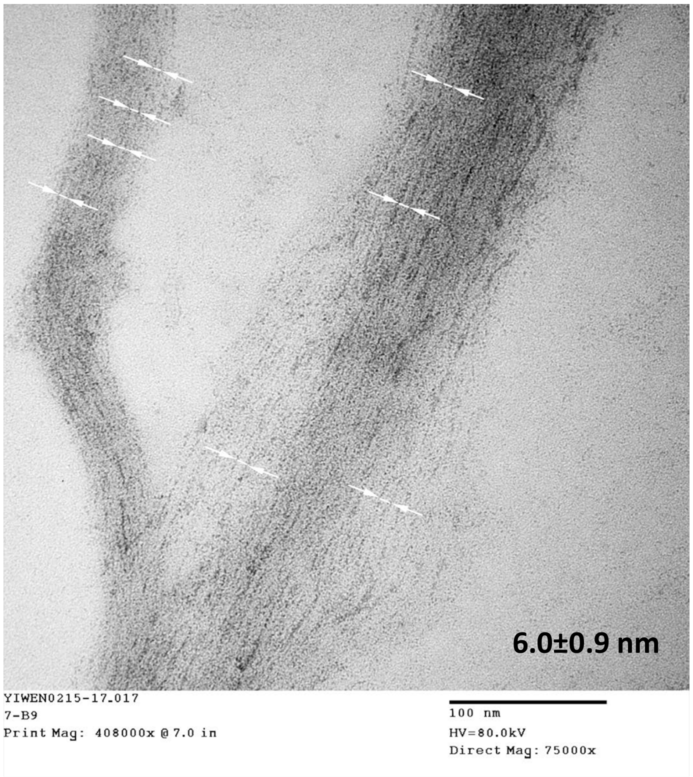

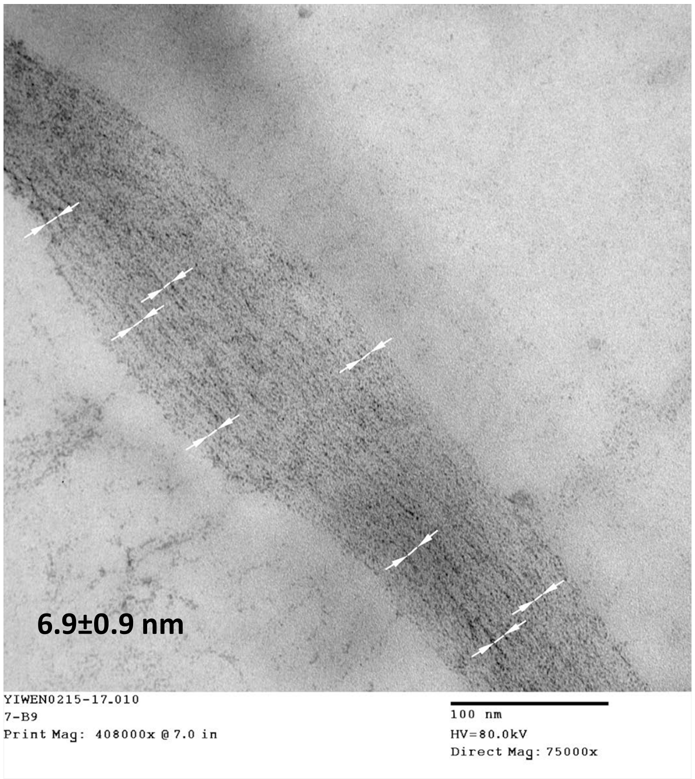

Figure S8. RNT diameter measurements from high resolution TEM data **GC12** (top) and **GC18** (bottom).

NMR Spectra

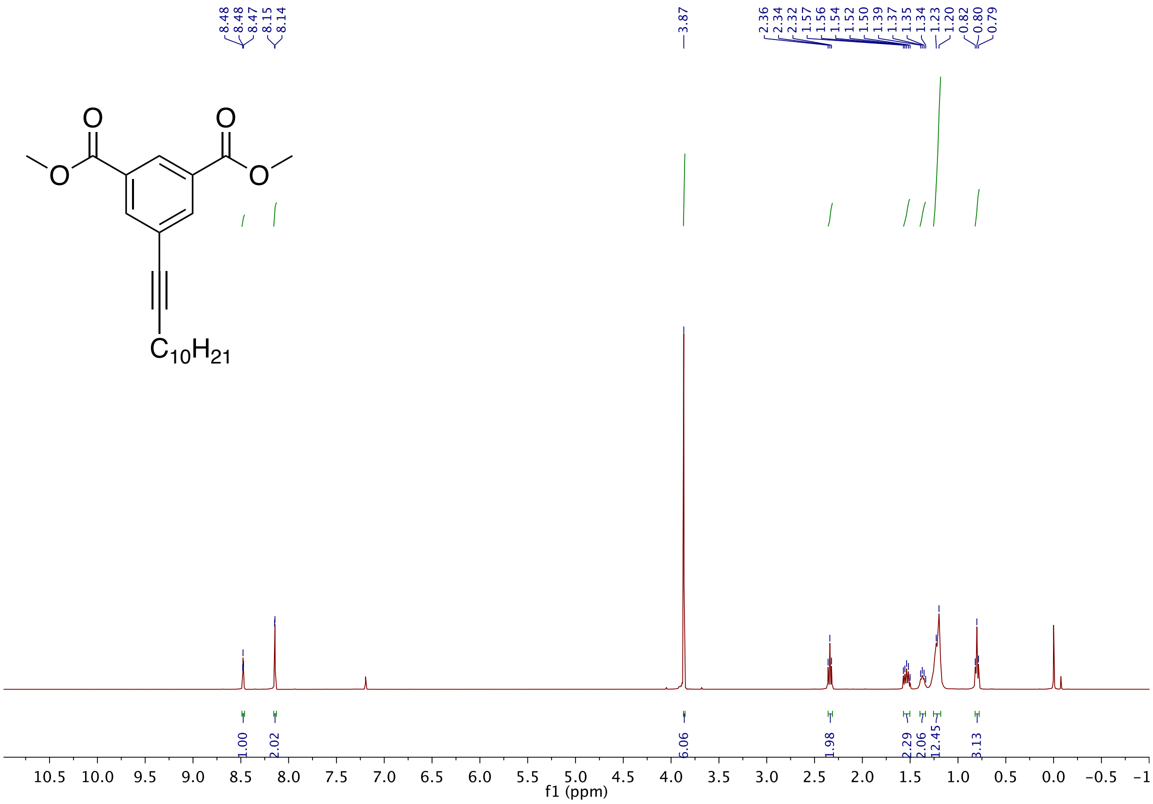
O
O
O
O
C10H21
8.48
8.48
8.47
8.15
8.14
3.87
2.36
2.34
2.32
1.57
1.56
1.54
1.52
1.50
1.39
1.37
1.35
1.34
1.23
1.20
0.82
0.80
0.79
1.00
2.02
6.06
1.98
2.29
2.06
12.45
3.13
10.5 10.0 9.5 9.0 8.5 8.0 7.5 7.0 6.5 6.0 5.5 5.0 4.5 4.0 3.5 3.0 2.5 2.0 1.5 1.0 0.5 0.0 −0.5 −1
f1 (ppm)

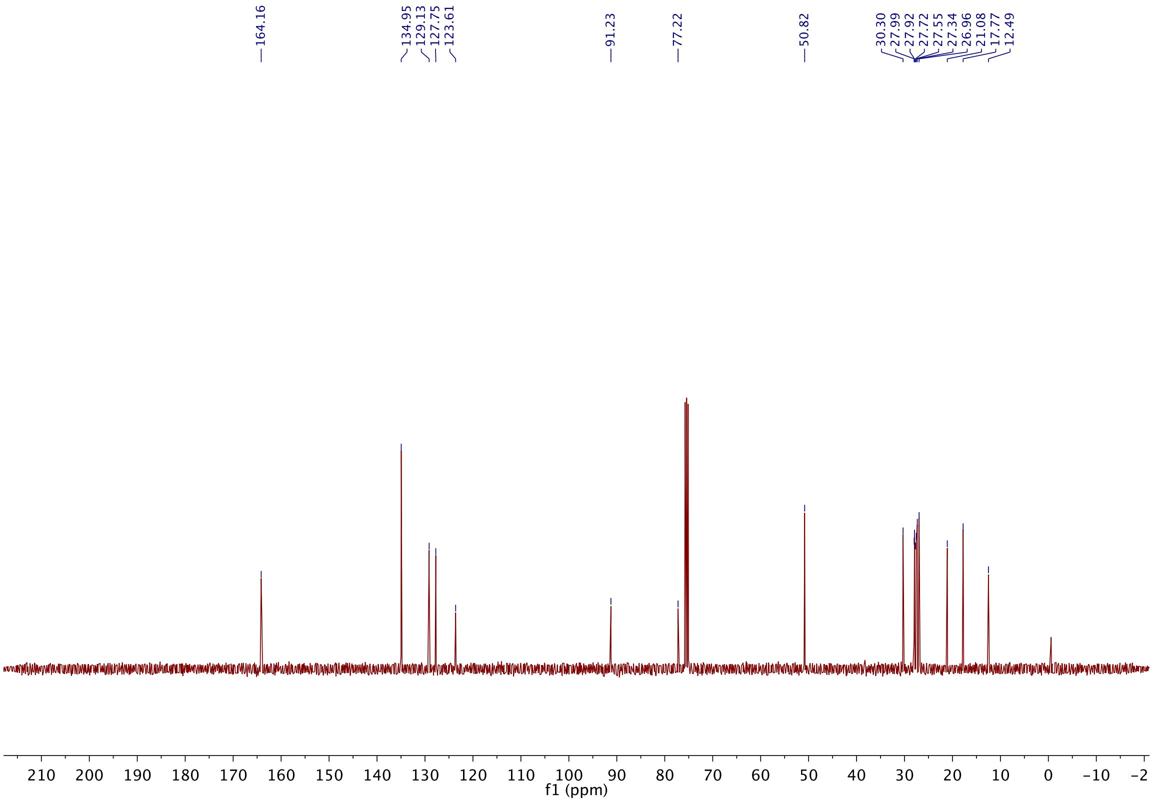
164.16
134.95
129.13
127.75
123.61
91.23
77.22
50.82
30.30
27.99
27.92
27.72
27.55
27.34
26.96
21.08
17.77
12.49
210 200 190 180 170 160 150 140 130 120 110 100 90 80 70 60 50 40 30 20 10 0 −10 −2
f1 (ppm)

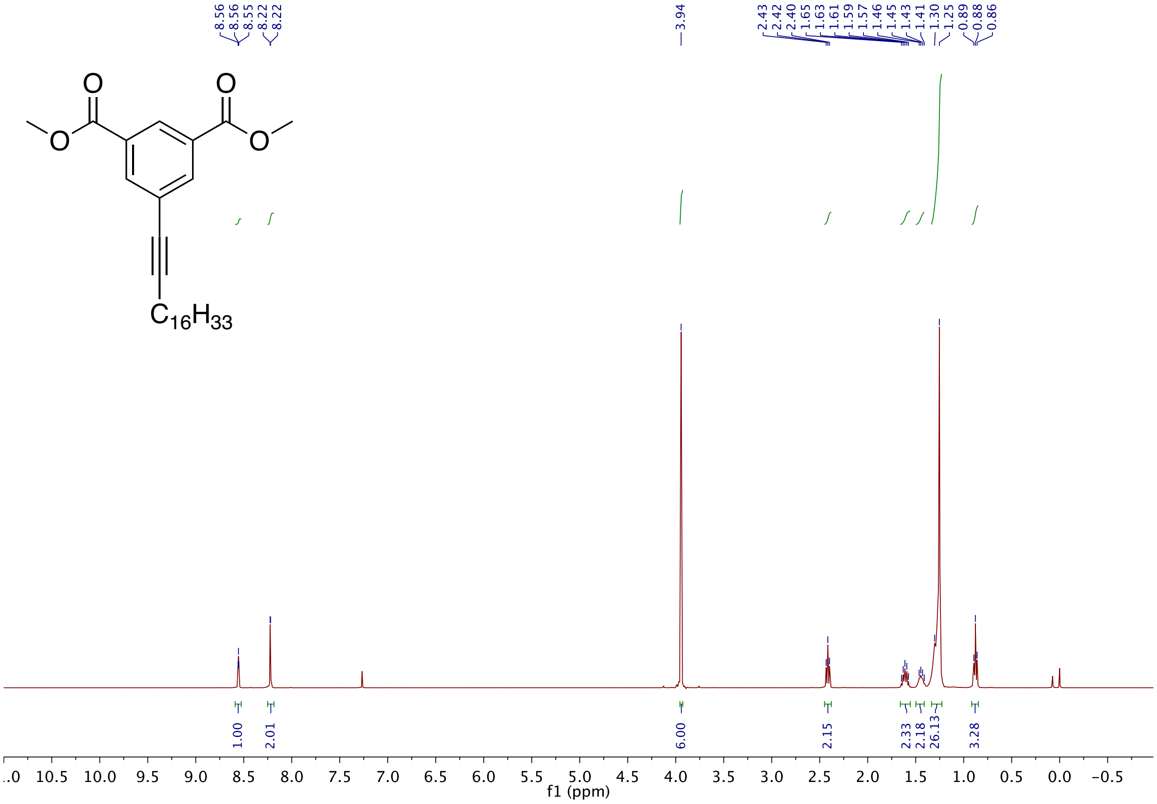

8.56
8.56
8.55
8.22
8.22
3.94
2.43
2.42
2.40
1.65
1.63
1.61
1.59
1.57
1.46
1.45
1.43
1.41
1.30
1.25
0.89
0.88
0.86
O
O
O
O
$C_{16}H_{33}$
1.00
2.01
6.00
2.15
2.33
2.18
26.13
3.28
f1 (ppm)

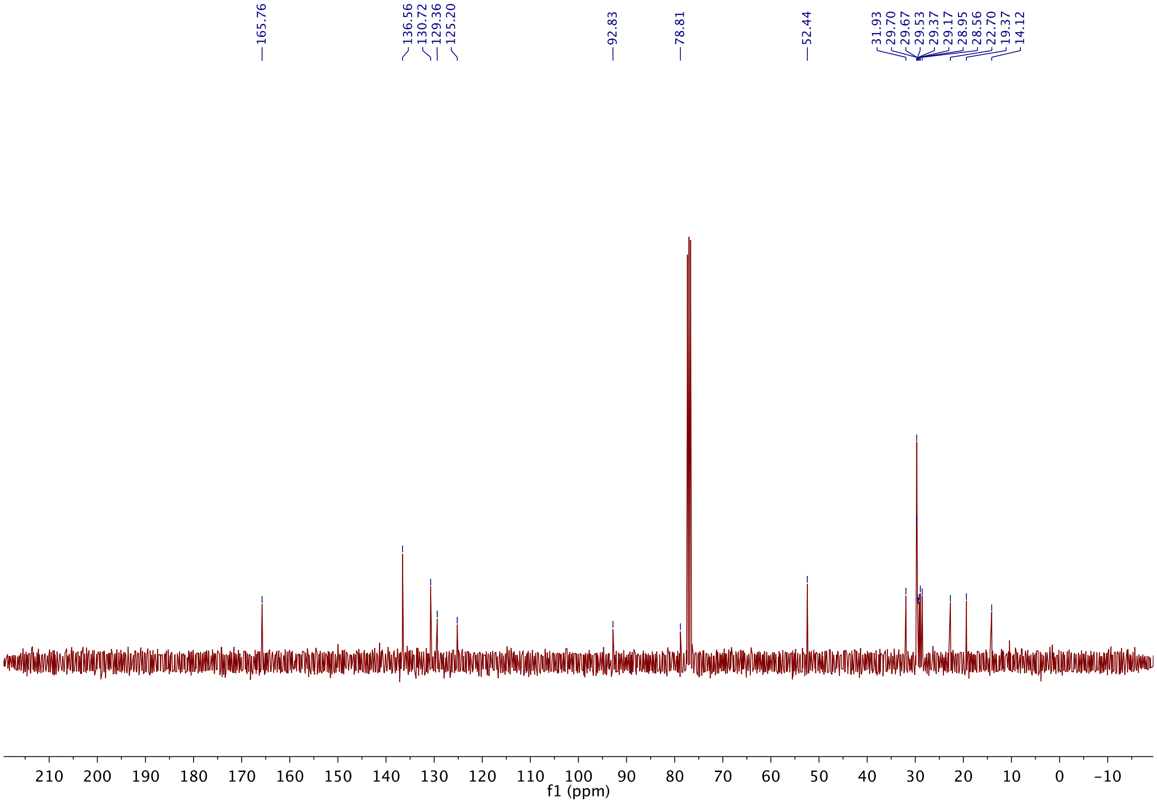

165.76
136.56
130.72
129.36
125.20
92.83
78.81
52.44
31.93
29.70
29.67
29.53
29.37
29.17
28.95
28.56
22.70
19.37
14.12
f1 (ppm)

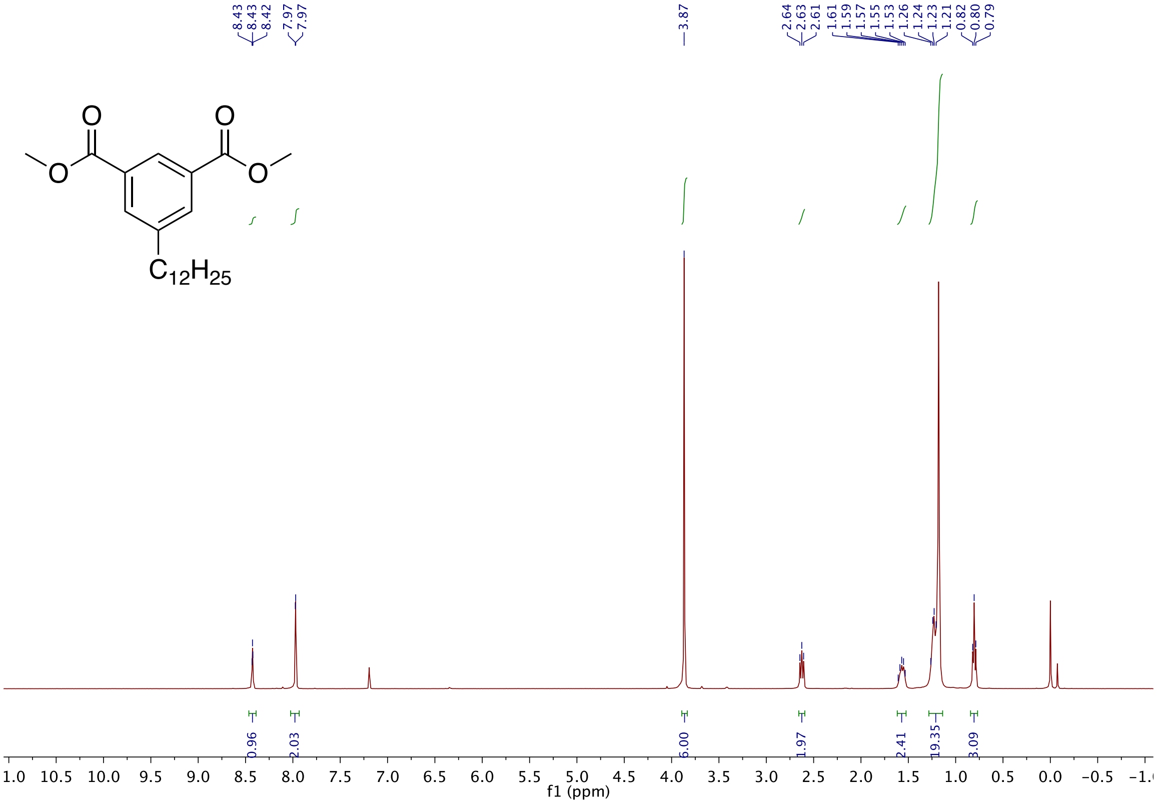
8.43
8.43
8.42
7.97
7.97
3.87
2.64
2.63
2.61
1.61
1.59
1.57
1.55
1.53
1.26
1.24
1.23
1.21
0.82
0.80
0.79
O
O
O
O
$C_{12}H_{25}$
0.96
2.03
6.00
1.97
2.41
19.35
3.09
f1 (ppm)

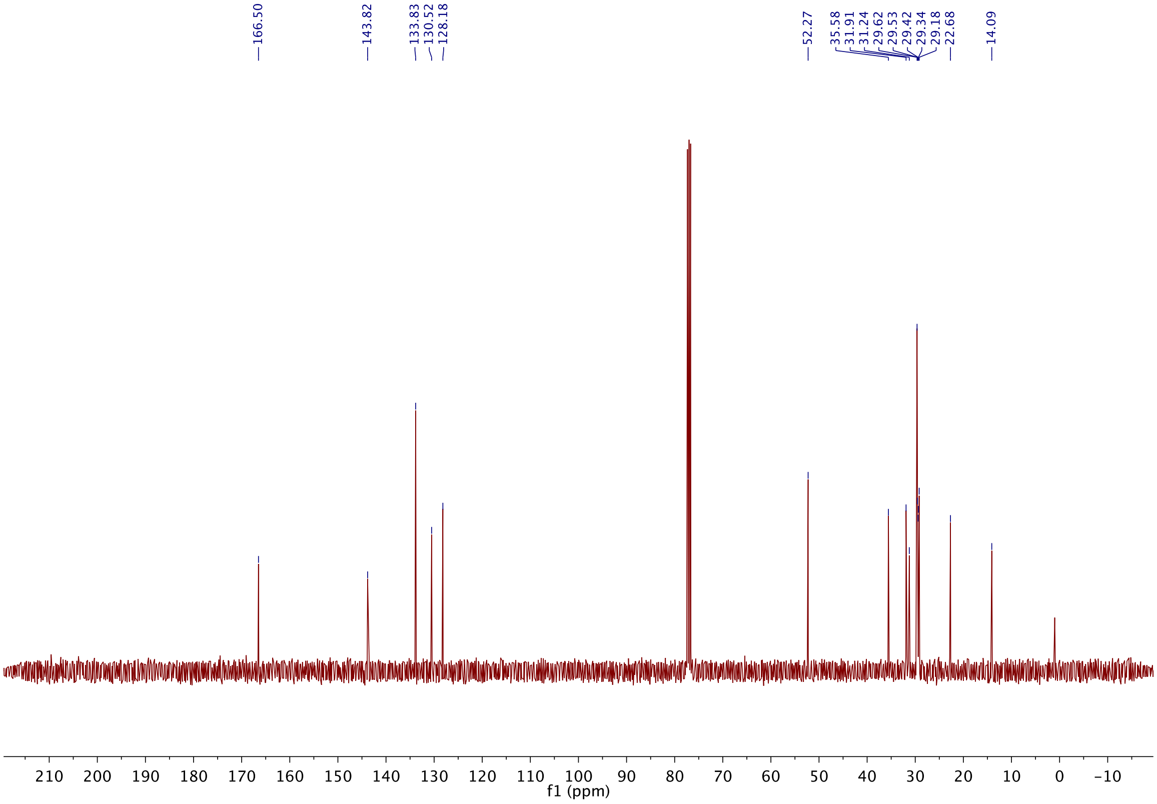
166.50
143.82
133.83
130.52
128.18
52.27
35.58
31.91
31.24
29.62
29.53
29.42
29.34
29.18
22.68
14.09
f1 (ppm)

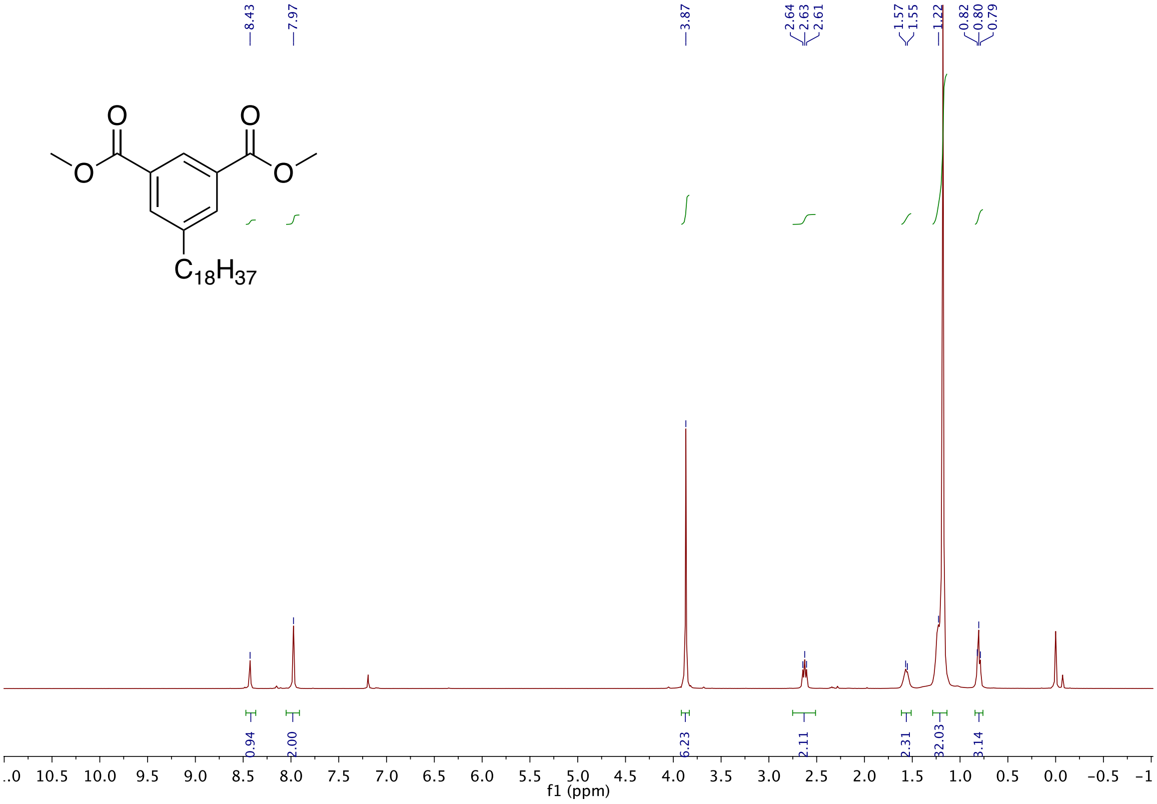

O
O
O
O
C18H37
8.43
7.97
3.87
2.64
2.63
2.61
1.57
1.55
1.22
0.82
0.80
0.79
0.94
2.00
6.23
2.11
2.31
32.03
3.14
f1 (ppm)

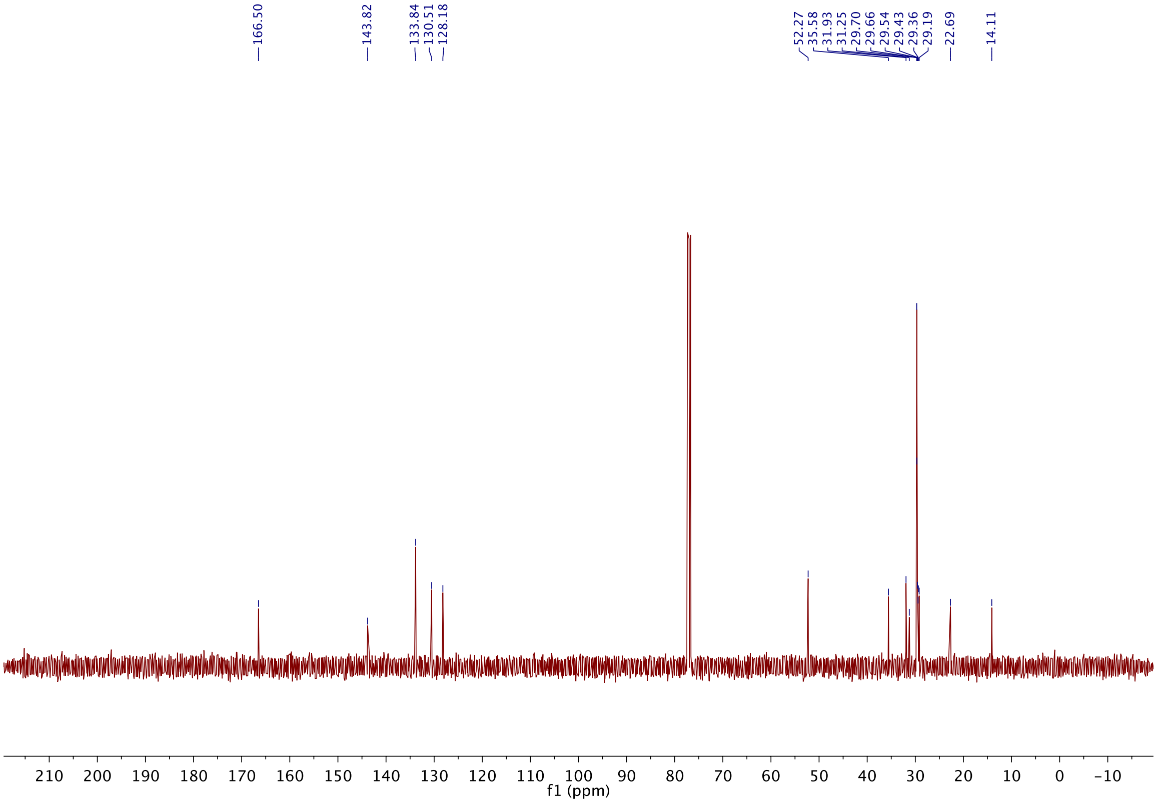

166.50
143.82
133.84
130.51
128.18
52.27
35.58
31.93
31.25
29.70
29.66
29.54
29.43
29.36
29.19
22.69
14.11
f1 (ppm)

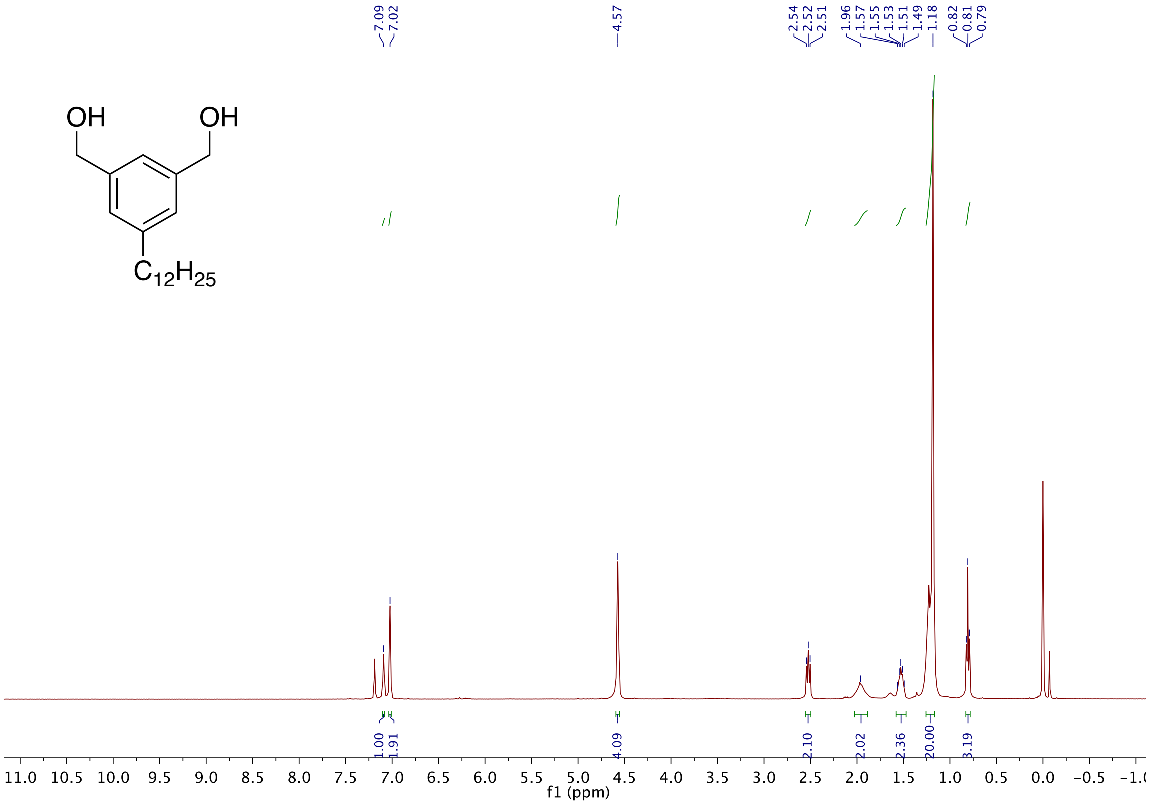
OH
OH
$C_{12}H_{25}$
7.09
7.02
4.57
2.54
2.52
2.51
1.96
1.57
1.55
1.53
1.51
1.49
1.18
0.82
0.81
0.79
1.00
1.91
4.09
2.10
2.02
2.36
20.00
3.19
11.0 10.5 10.0 9.5 9.0 8.5 8.0 7.5 7.0 6.5 6.0 5.5 5.0 4.5 4.0 3.5 3.0 2.5 2.0 1.5 1.0 0.5 0.0 −0.5
f1 (ppm)

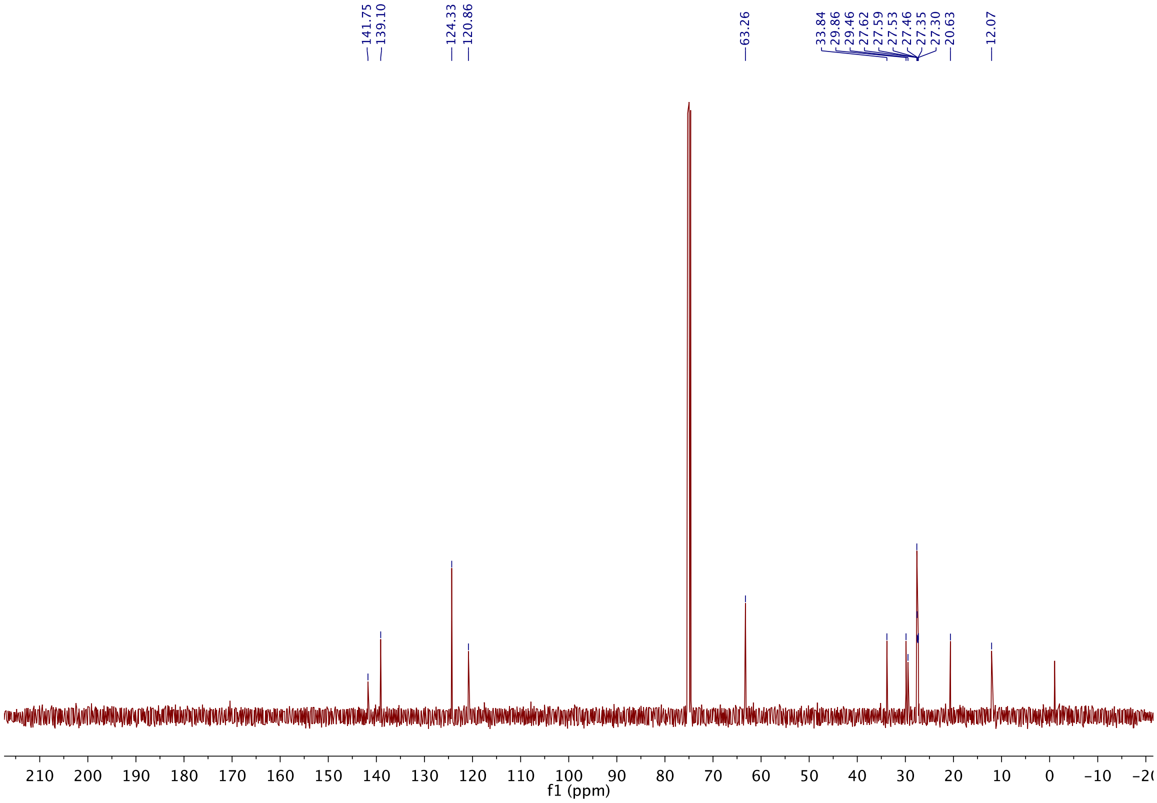
141.75
139.10
124.33
120.86
63.26
33.84
29.86
29.46
27.62
27.59
27.53
27.46
27.35
27.30
20.63
12.07
210 200 190 180 170 160 150 140 130 120 110 100 90 80 70 60 50 40 30 20 10 0 −10
f1 (ppm)

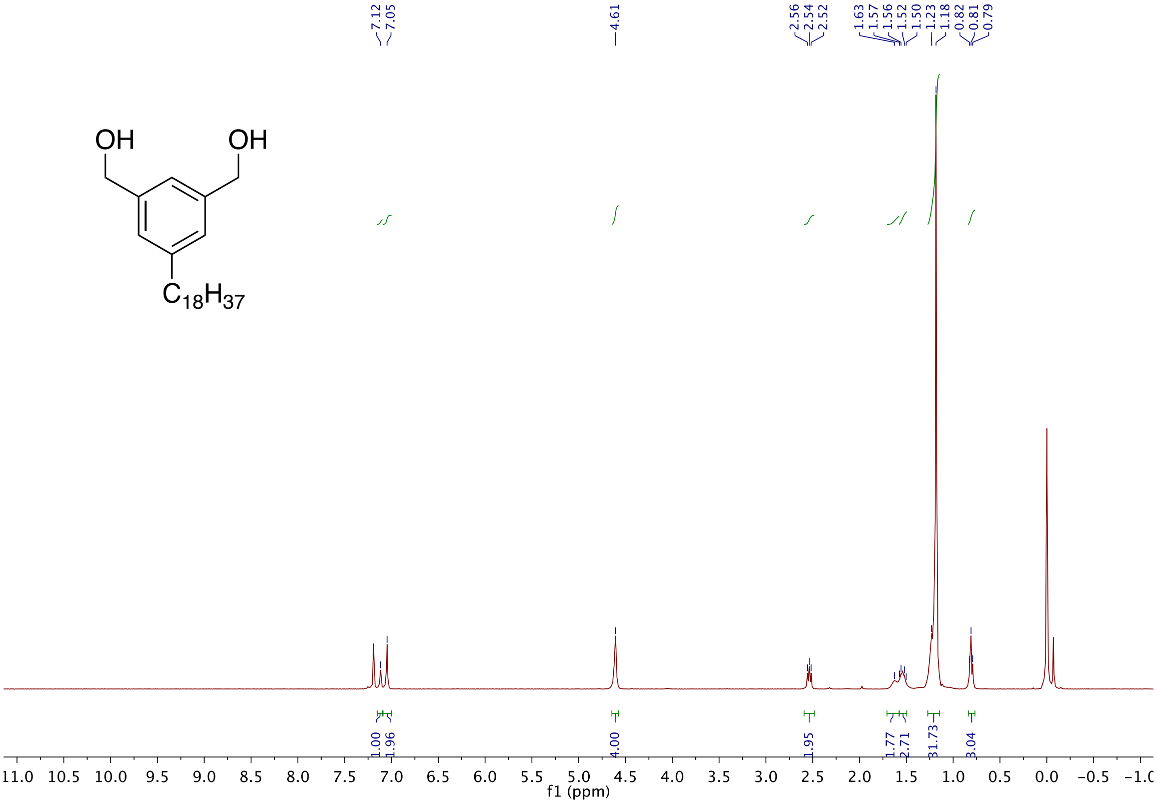
7.12
7.05
4.61
2.56
2.54
2.52
1.63
1.57
1.56
1.52
1.50
1.23
1.18
0.82
0.81
0.79
OH
OH
$C_{18}H_{37}$
1.00
1.96
4.00
1.95
1.77
2.71
31.73
3.04
f1 (ppm)

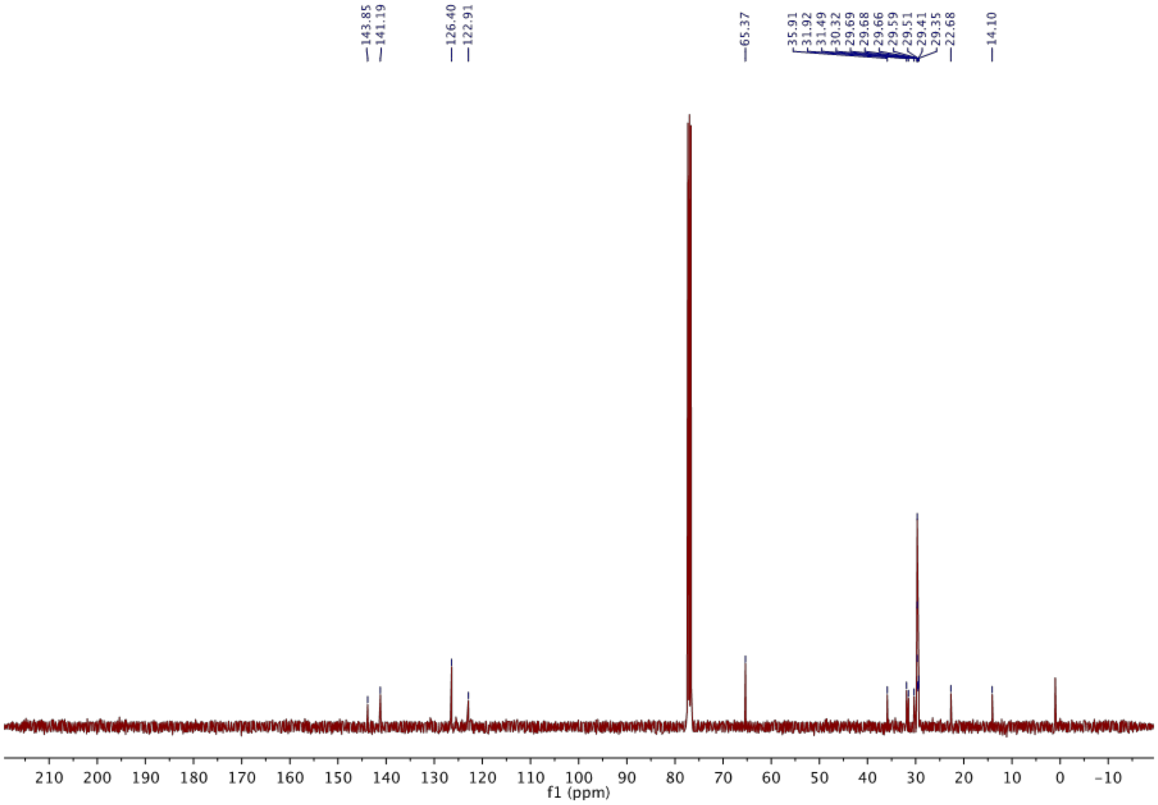
143.85
141.19
126.40
122.91
65.37
35.91
31.92
31.49
30.32
29.69
29.68
29.66
29.59
29.51
29.41
29.35
22.68
14.10
f1 (ppm)

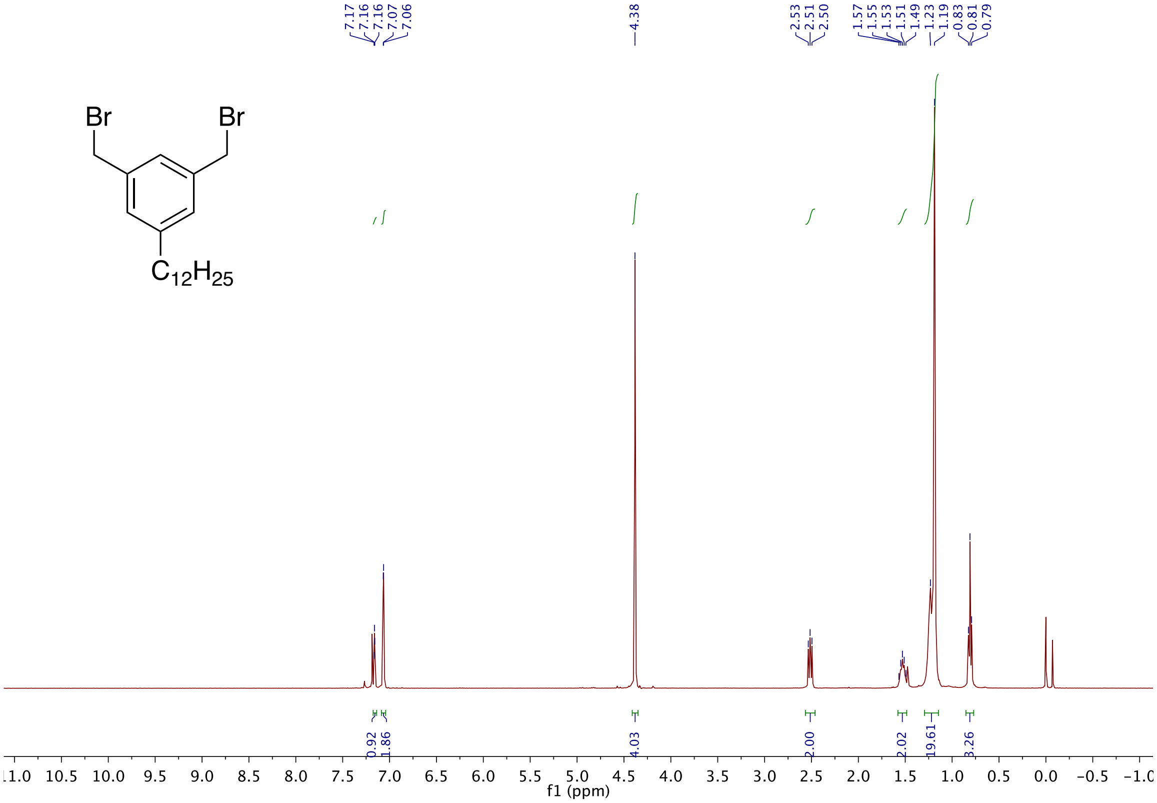
Br
Br
$C_{12}H_{25}$
7.17
7.16
7.16
7.07
7.06
4.38
2.53
2.51
2.50
1.57
1.55
1.53
1.51
1.49
1.23
1.19
0.83
0.81
0.79
0.92
1.86
4.03
2.00
2.02
19.61
3.26
11.0 10.5 10.0 9.5 9.0 8.5 8.0 7.5 7.0 6.5 6.0 5.5 5.0 4.5 4.0 3.5 3.0 2.5 2.0 1.5 1.0 0.5 0.0 −0.5 −1.0
f1 (ppm)

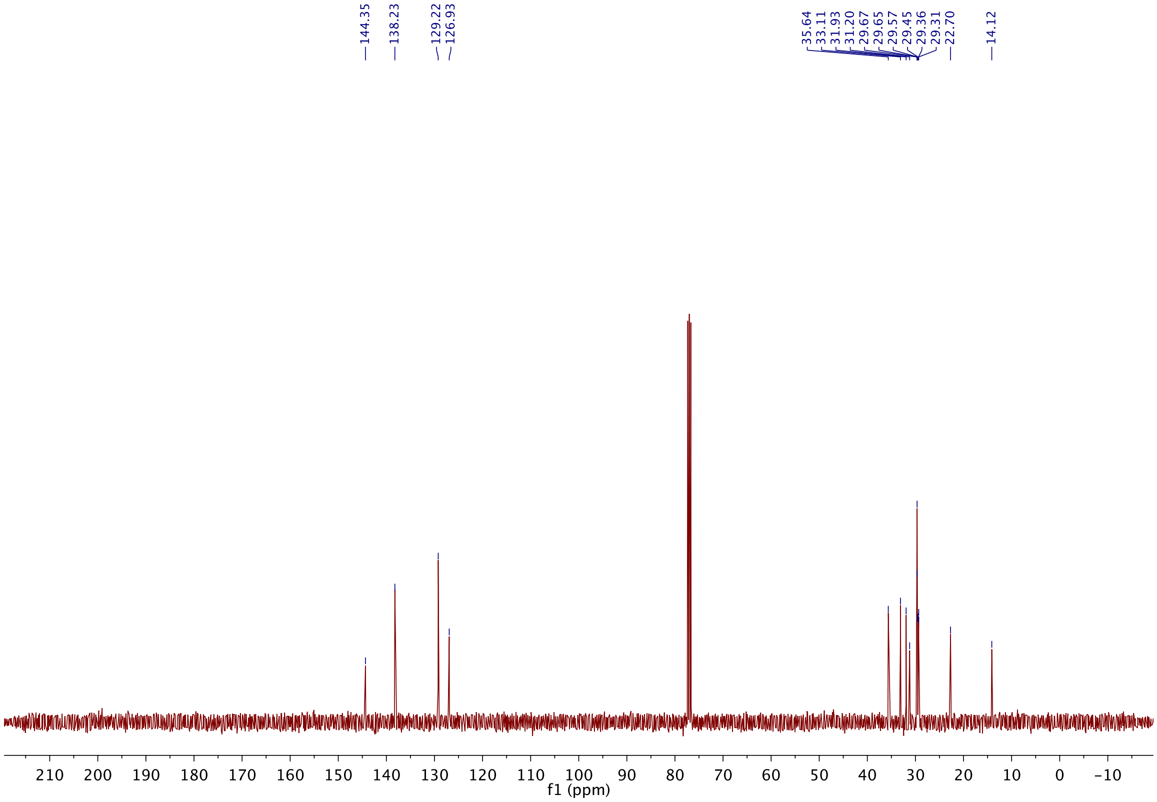
144.35
138.23
129.22
126.93
35.64
33.11
31.93
31.20
29.67
29.65
29.57
29.45
29.36
29.31
22.70
14.12
210 200 190 180 170 160 150 140 130 120 110 100 90 80 70 60 50 40 30 20 10 0 −10
f1 (ppm)

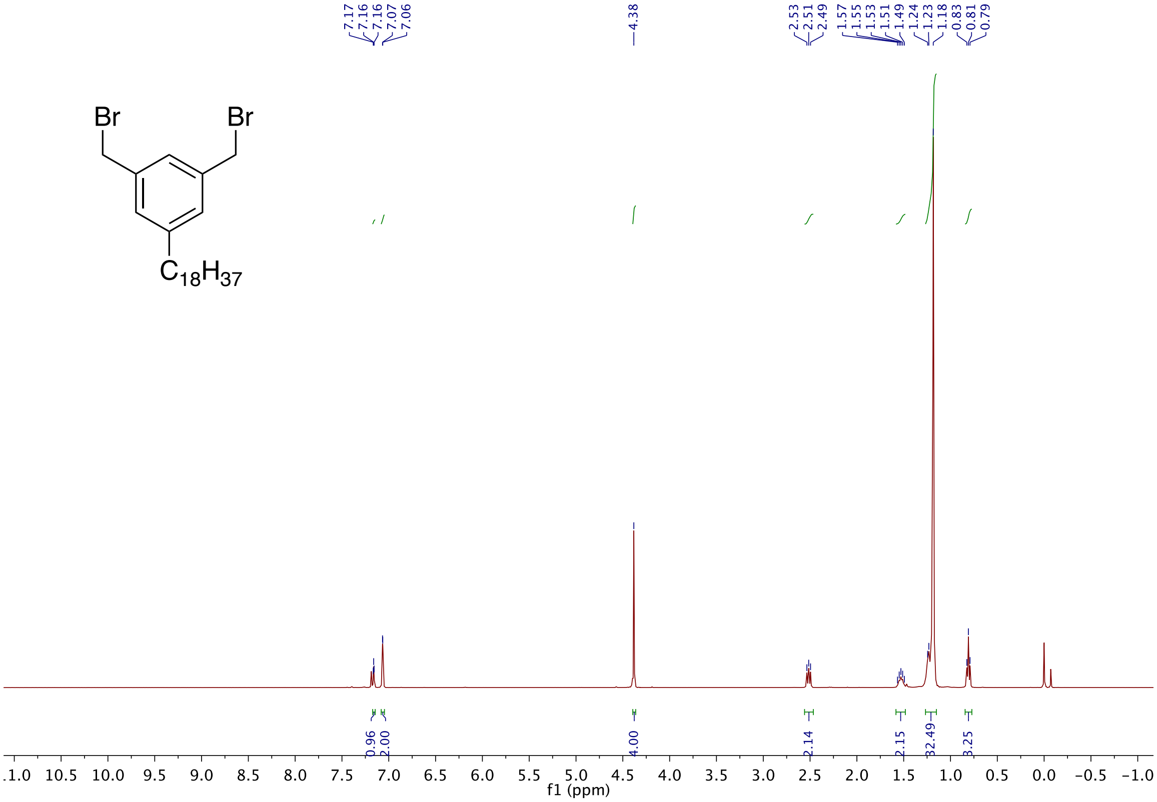
Br
Br
$C_{18}H_{37}$
7.17
7.16
7.16
7.07
7.06
4.38
2.53
2.51
2.49
1.57
1.55
1.53
1.51
1.49
1.24
1.23
1.18
0.83
0.81
0.79
0.96
2.00
4.00
2.14
2.15
32.49
3.25
f1 (ppm)

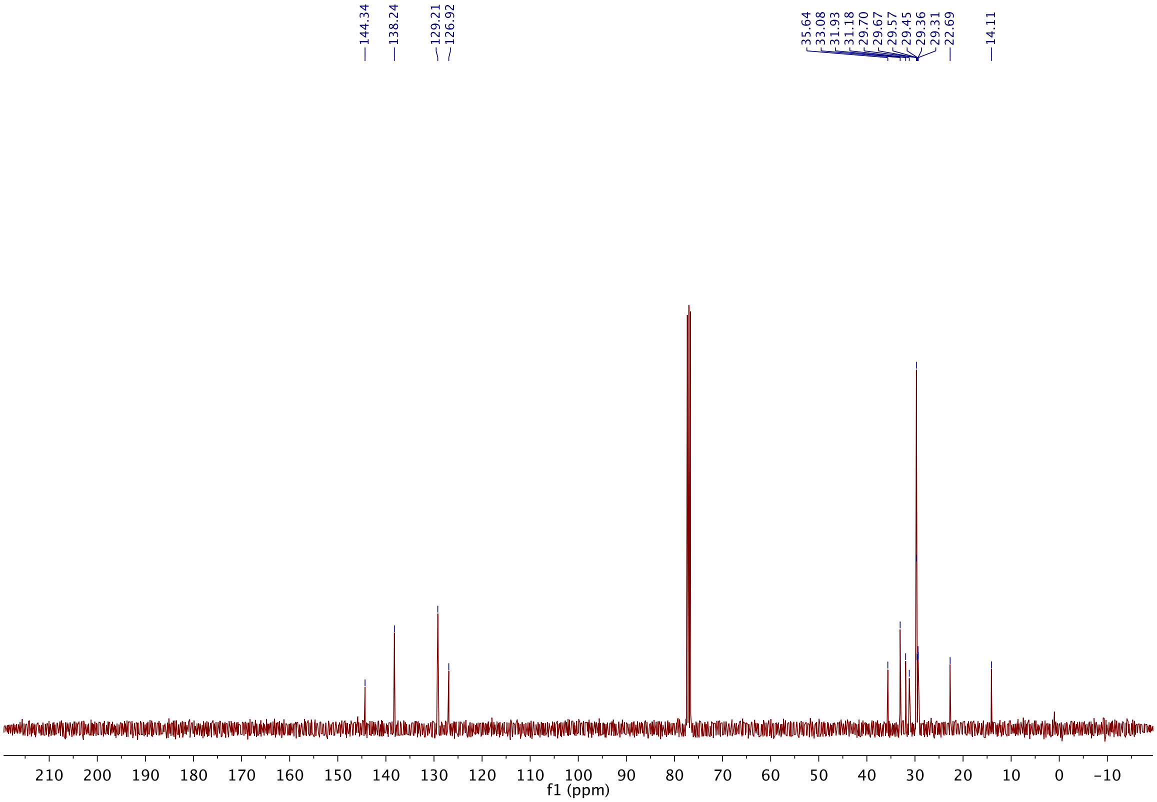
144.34
138.24
129.21
126.92
35.64
33.08
31.93
31.18
29.70
29.67
29.57
29.45
29.36
29.31
22.69
14.11
f1 (ppm)

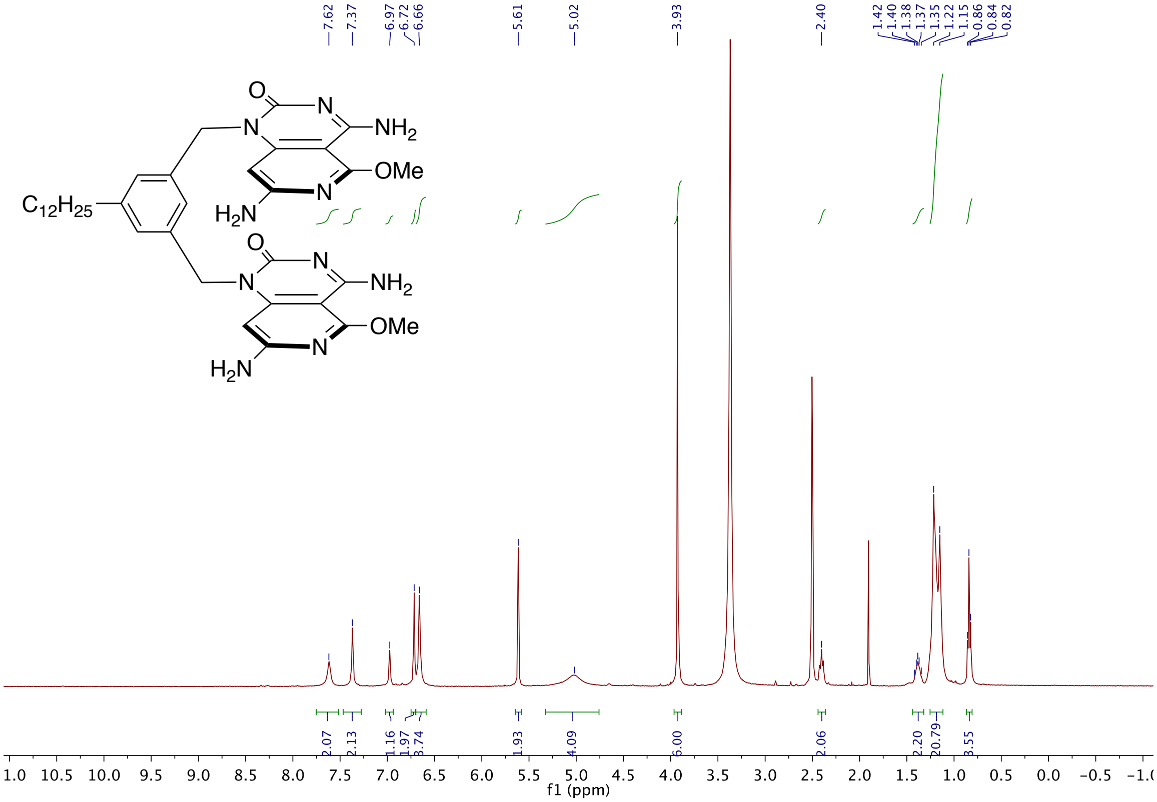
7.62
7.37
6.97
6.72
6.66
5.61
5.02
3.93
2.40
1.42
1.40
1.38
1.37
1.35
1.22
1.15
0.86
0.84
0.82
O
N
NH2
N
OMe
N
C12H25
H2N
O
N
N
NH2
OMe
N
H2N
2.07
2.13
1.16
1.97
3.74
1.93
4.09
6.00
2.06
2.20
20.79
3.55
f1 (ppm)

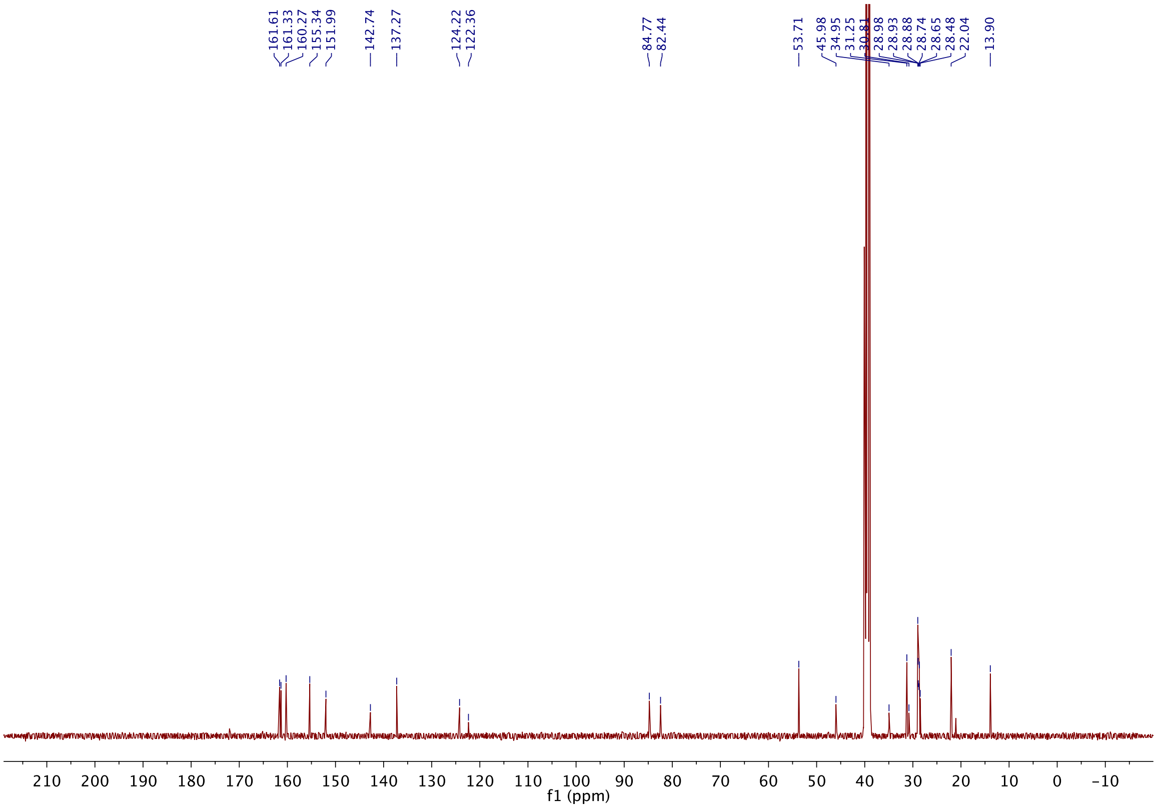
161.61
161.33
160.27
155.34
151.99
142.74
137.27
124.22
122.36
84.77
82.44
53.71
45.98
34.95
31.25
30.81
28.98
28.93
28.88
28.74
28.65
28.48
22.04
13.90
f1 (ppm)

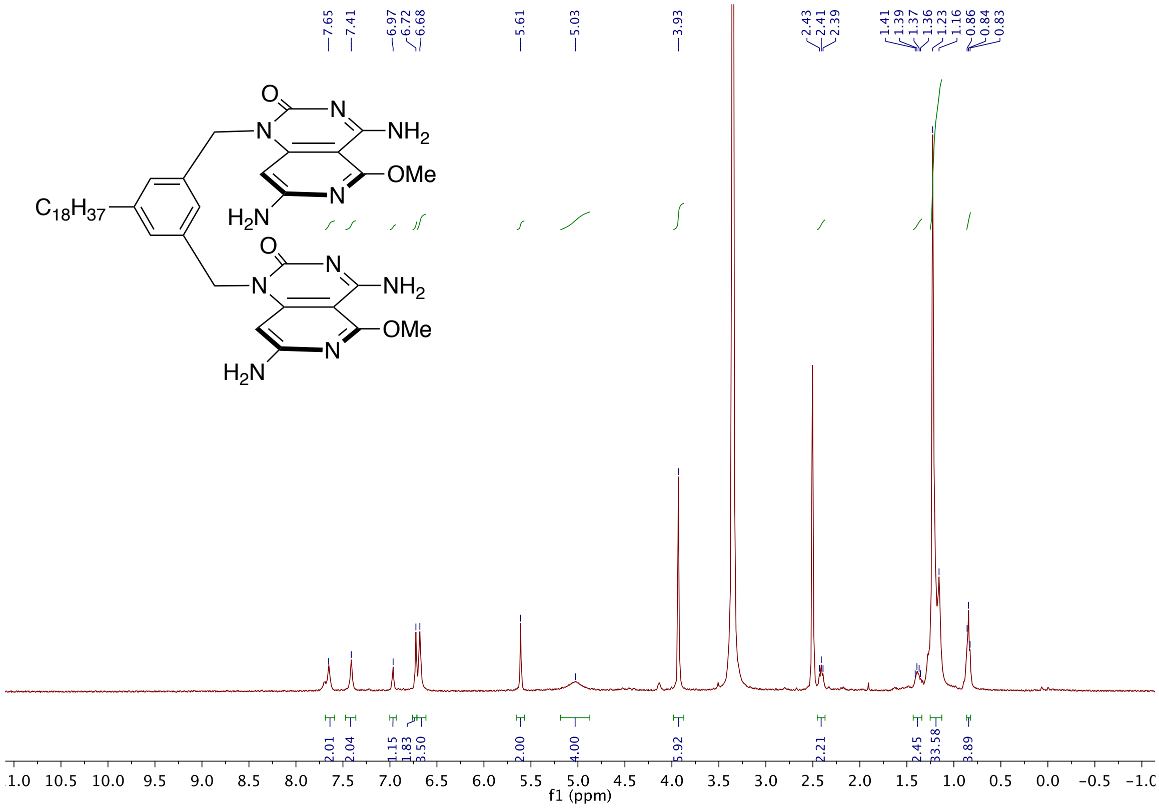
7.65
7.41
6.97
6.72
6.68
5.61
5.03
3.93
2.43
2.41
2.39
1.41
1.39
1.37
1.36
1.23
1.16
0.86
0.84
0.83
O
N
N
NH2
OMe
H2N
C18H37
2.01
2.04
1.15
1.83
3.50
2.00
4.00
5.92
2.21
2.45
33.58
3.89
1.0 10.5 10.0 9.5 9.0 8.5 8.0 7.5 7.0 6.5 6.0 5.5 5.0 4.5 4.0 3.5 3.0 2.5 2.0 1.5 1.0 0.5 0.0 −0.5 −1.0
f1 (ppm)

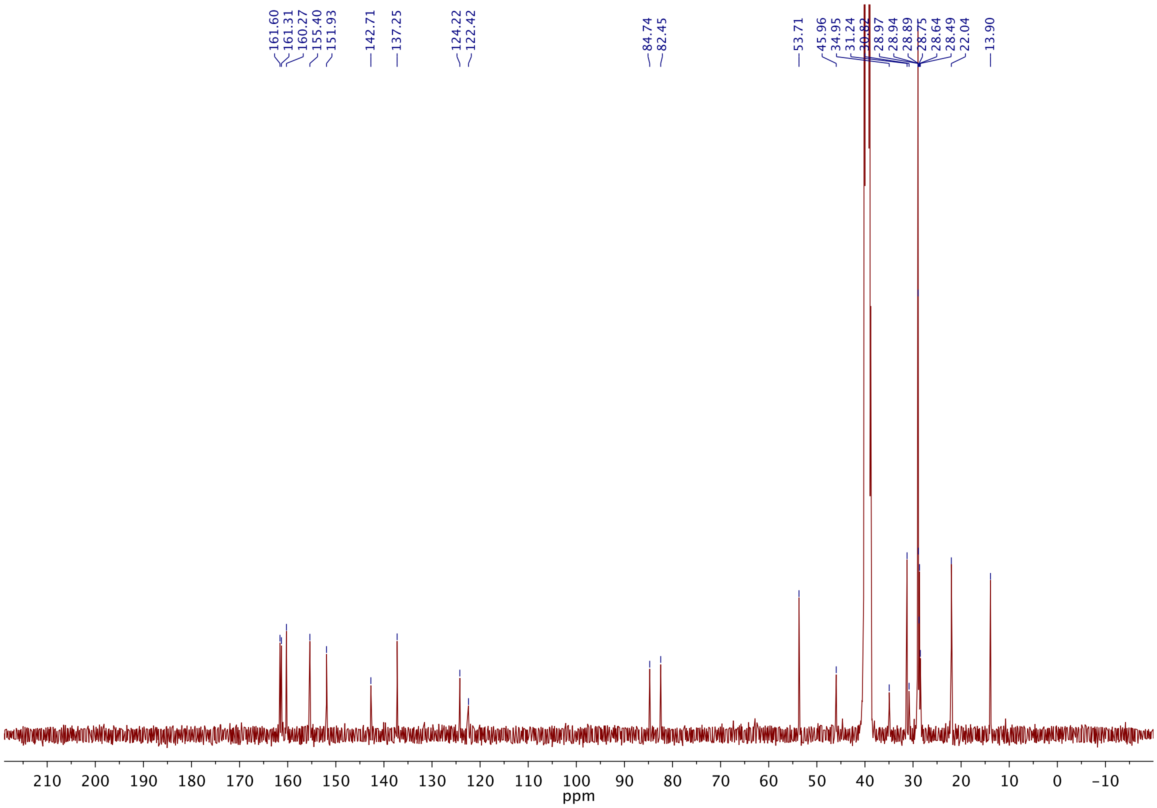
161.60
161.31
160.27
155.40
151.93
142.71
137.25
124.22
122.42
84.74
82.45
53.71
45.96
34.95
31.24
30.82
28.97
28.94
28.89
28.75
28.64
28.49
22.04
13.90
210 200 190 180 170 160 150 140 130 120 110 100 90 80 70 60 50 40 30 20 10 0 −10
ppm

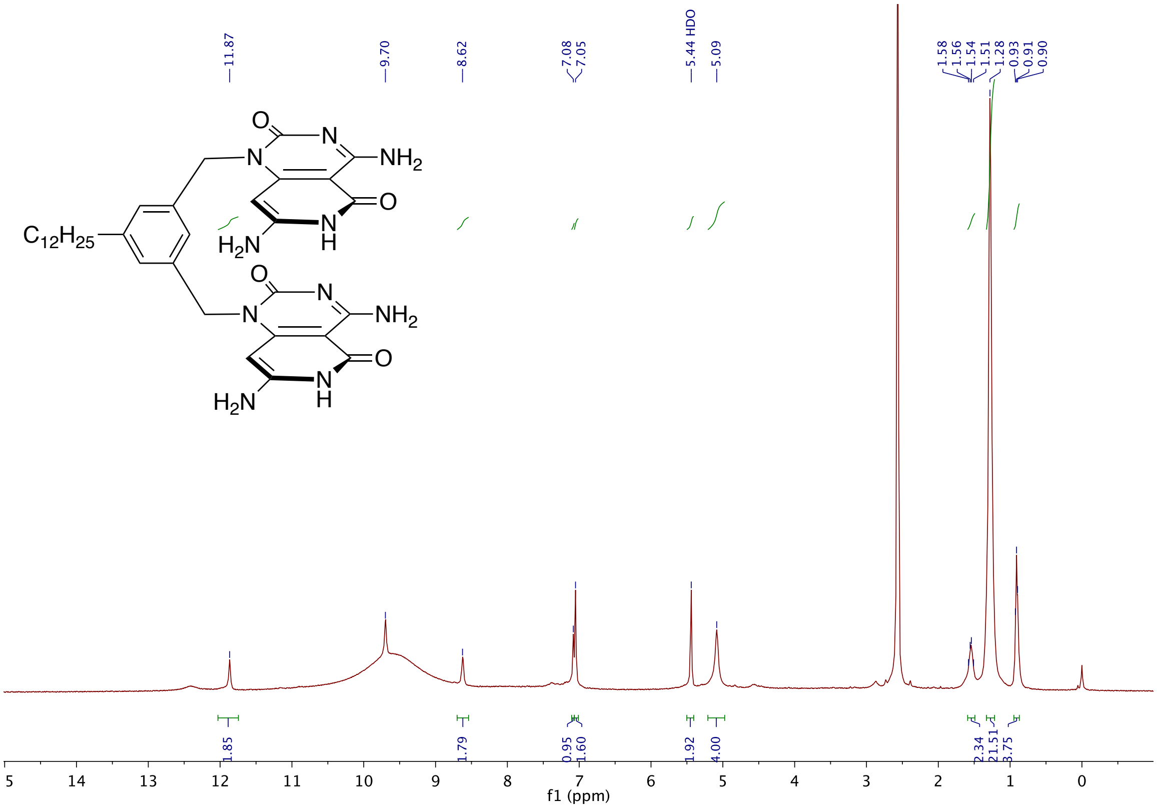
11.87
9.70
8.62
7.08
7.05
5.44 HDO
5.09
1.58
1.56
1.54
1.51
1.28
0.93
0.91
0.90
NH2
H2N
C12H25
1.85
1.79
0.95
1.60
1.92
4.00
2.34
21.51
3.75
f1 (ppm)

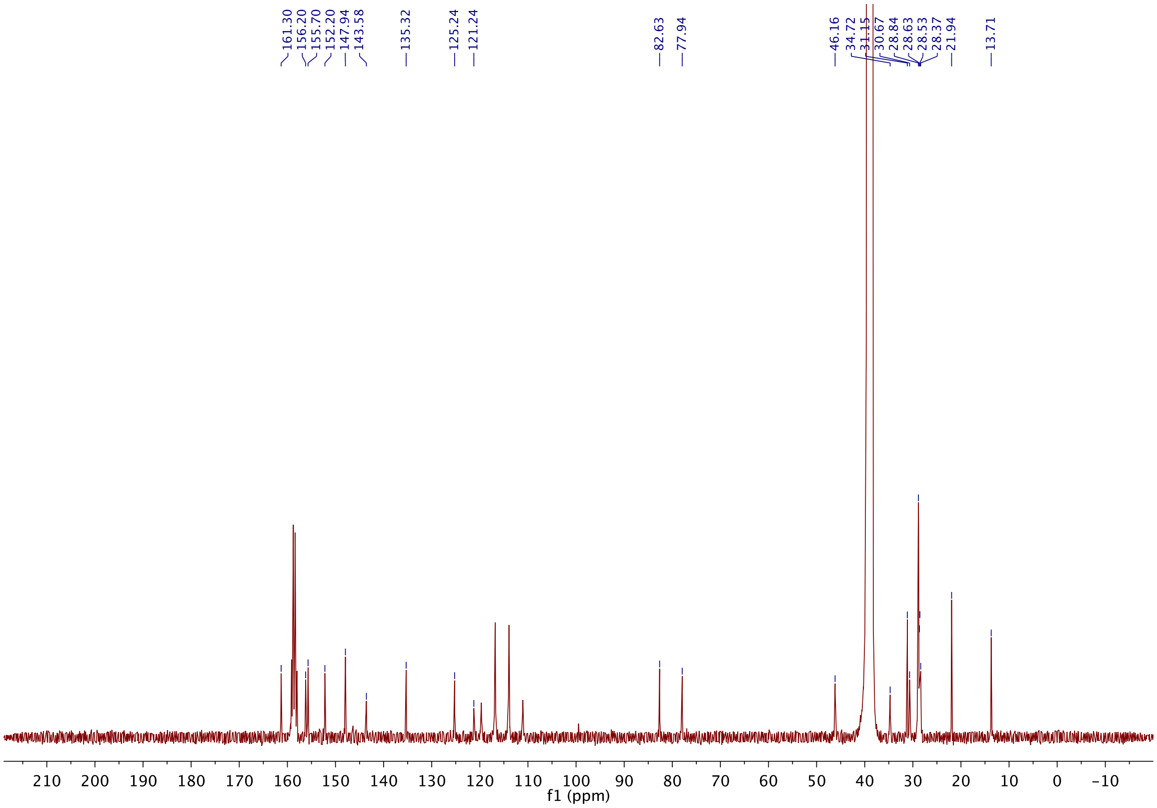
161.30
156.20
155.70
152.20
147.94
143.58
135.32
125.24
121.24
82.63
77.94
46.16
34.72
31.15
30.67
28.84
28.63
28.53
28.37
21.94
13.71
f1 (ppm)

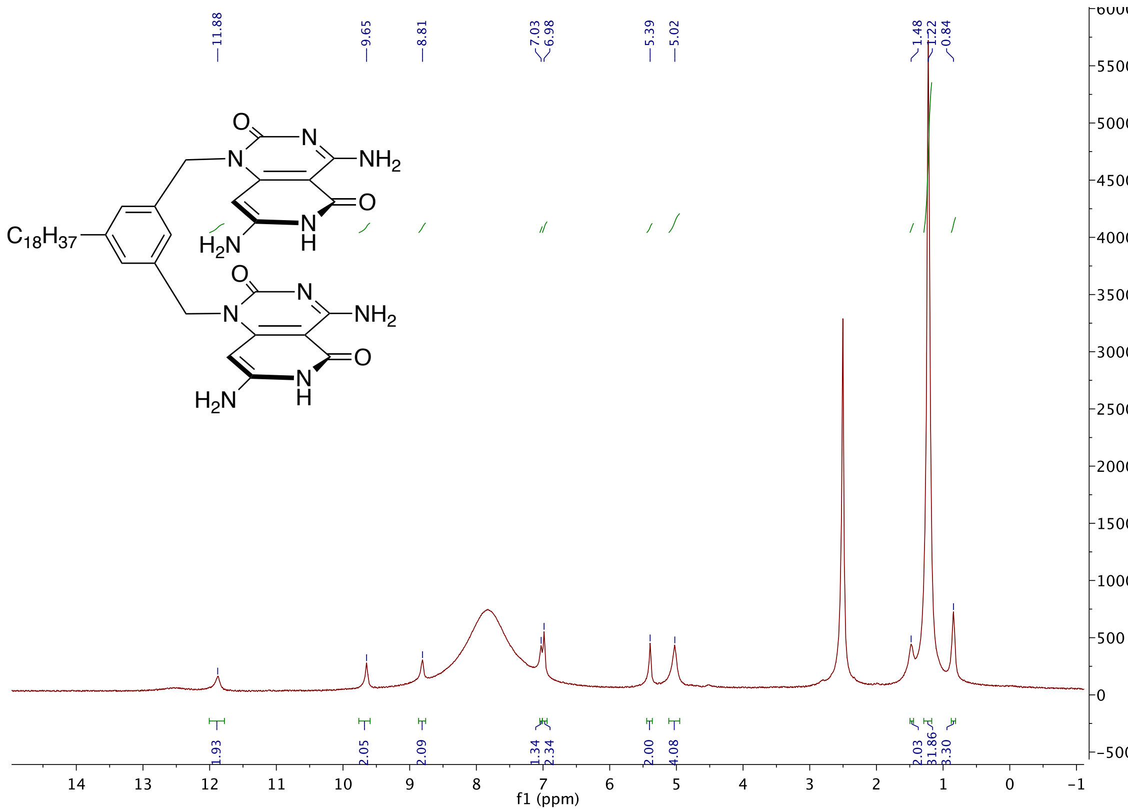
11.88
9.65
8.81
7.03
6.98
5.39
5.02
1.48
1.22
0.84
1.93
2.05
2.09
1.34
2.34
2.00
4.08
2.03
31.86
3.30
$C_{18}H_{37}$
NH_2
H_2N
f1 (ppm)

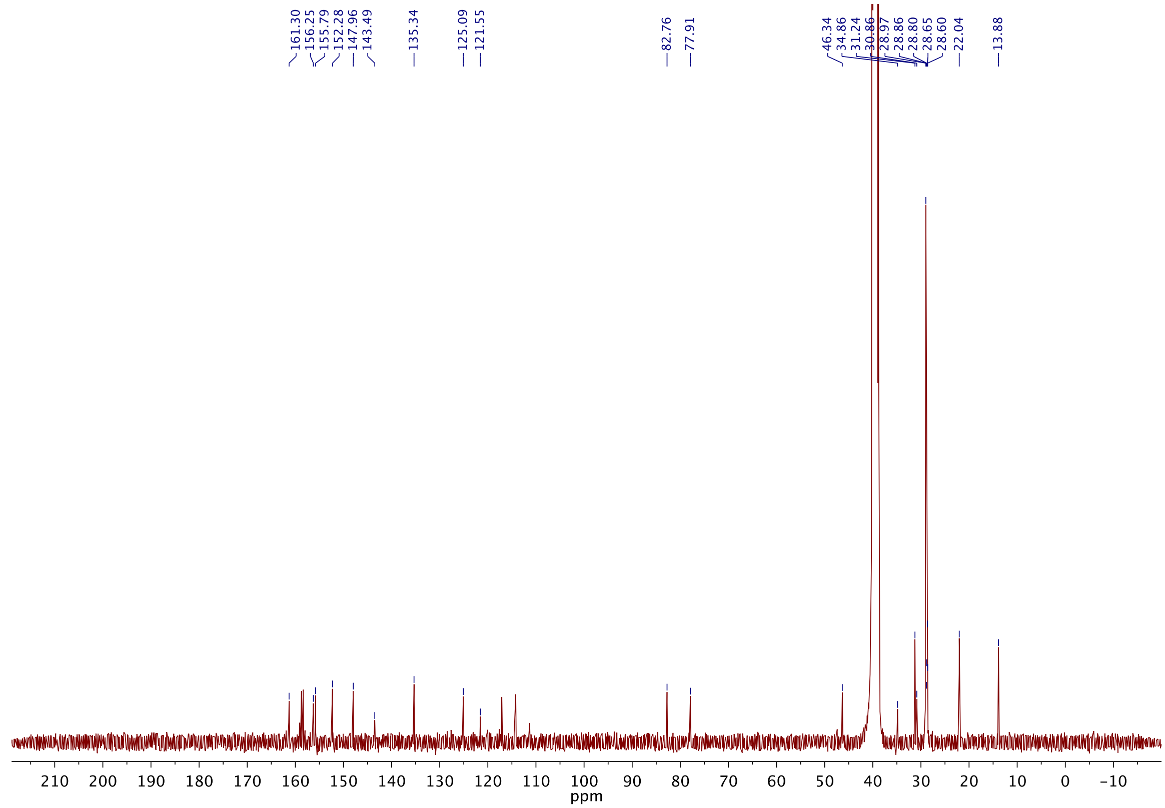
161.30
156.25
155.79
152.28
147.96
143.49
135.34
125.09
121.55
82.76
77.91
46.34
34.86
31.24
30.86
28.97
28.86
28.80
28.65
28.60
22.04
13.88
ppm

Cartesian Coordinates and Energies

The calculated total energies based on OPLS3 force field, their breakdown, and their relative energies are given in the below table.

**Structure**	**GCDR12**								
**Number of Motifs**	**Free Energy (kJ/mol)**	**Stretch Energy (kJ/mol)**	**Bend Energy (kJ/mol)**	**Torsional Energy (kJ/mol)**	**Improper Torsional Energy (kJ/mol)**	**Van der Waals Energy (kJ/mol)**	**Electrostatic Energy (kJ/mol)**	**Solvation Energy (kJ/mol)**	**Relative Free Energy (kJ/mol)**
1	-858.474	50.932	150.806	8.207	0.22	267.921	-1157.695	-178.863	0
6	-5700.074	305.601	904.836	49.242	1.322	1656.446	-8121.848	-495.674	-549.23
12	-11760.74	612.838	1820.017	61.304	2.202	2929.178	-16241.396	-944.881	-1459.048
18	-17824.07	920.095	2735.117	72.753	3.09	4202.473	-24361.916	-1395.682	-2371.538
24	-23887.17	1227.188	3650.316	84.545	3.944	5475.893	-32484.352	-1844.708	-3283.796
30	-29909.79	1532.819	4555.155	133.783	5.263	6748.186	-40609.199	-2275.795	-4155.567
**Structure**	**GCDR18**								
**Number of Motifs**	**Free Energy (kJ/mol)**	**Stretch Energy (kJ/mol)**	**Bend Energy (kJ/mol)**	**Torsional Energy (kJ/mol)**	**Improper Torsional Energy (kJ/mol)**	**Van der Waal Energy (kJ/mol)**	**Electrostatic Energy (kJ/mol)**	**Solvation Energy (kJ/mol)**	**Relative Free Energy (kJ/mol)**
1	-826.288	51.932	152.962	10.713	0.271	268.356	-1130.204	-180.318	0
6	-5485.055	311.59	917.775	64.274	1.629	1662.42	-7911.767	-530.976	-527.327
12	-11324.53	622.68	1837.917	110.873	2.925	2939.813	-15835.64	-1003.101	-1409.076
18	-17164.91	933.77	2758.243	157.086	4.189	4217.527	-23759.441	-1476.286	-2291.728
24	-23002.94	1244.594	3677.156	207.895	5.564	5494.31	-31685.51	-1946.953	-3172.031
30	-28807.8	1556.18	4594.928	272.175	7.195	6770.709	-39594.047	-2414.933	-4019.155

References

1 Lee, J. R. I. *et al.* Cooperative Reorganization of Mineral and Template during Directed Nucleation of Calcium Carbonate. *J. Phys. Chem. C* **117**, 11076-11085 (2013).

2 Brousmiche, D. W. *et al.* Fluorescence resonance energy transfer in novel multiphoton absorbing dendritic structures. *J. Phys. Chem. B* **108**, 8592-8600 (2004).

3 Tran, V. M., Nguyen, T. K. N., Sorna, V., Loganathan, D. & Kuberan, B. Synthesis and Assessment of Glycosaminoglycan Priming Activity of Cluster-xylosides for Potential Use as Proteoglycan Mimetics. *ACS Chem. Biol.* **8**, 949-957 (2013).
